# Disentangling batch effects and biological signals across conditions in single-cell transcriptome

**DOI:** 10.1016/j.patter.2026.101638

**Published:** 2026-08-19

**Authors:** Shunta Sakaguchi, Masato Tsutsumi, Kentaro Nishi, Honda Naoki

**Affiliations:** 1 Nagoya University Graduate School of Medicine, Nagoya, Aichi 466-8550, Japan; 2 Graduate School of Integrated Life Science, Hiroshima University, Higashi-hiroshima, Hiroshima 739-8528, Japan; 3 Life Science Center for Survival Dynamics, Tsukuba Advanced Research Alliance (TARA), University of Tsukuba, Tsukuba, Ibaraki 305-8577, Japan; 4 Center for One Medicine Innovative Translational Research (COMIT), Nagoya University, Nagoya, Aichi 464-8601, Japan

**Keywords:** single-cell omics, batch correction, Deep learning

## Abstract

Batch effects, which arise due to technical variations across different experimental factors, pose a significant challenge for single-cell transcriptomic analysis. Although various batch correction methods have been developed to mitigate these effects, they often indiscriminately mix data from different batches, leading to the removal of biologically meaningful signals. This limitation hinders comparative analyses across multiple conditions, an essential aspect of scientific research. Recent approaches attempt to address this issue by mapping data to separate spaces, but they prevent direct comparisons between conditions. Here, we propose Kanade, a batch correction method based on a variational autoencoder. Kanade explicitly disentangles batch effects from biological signals by specializing latent variables for different types of information. Using both simulated and real datasets, we demonstrate that Kanade selectively corrects batch effects while preserving essential biological features, enabling more accurate comparative analyses in single-cell transcriptomics.

## Highlights

• Kanade separates batch effects from condition-specific biological signals
• Specialized latent variables enable selective removal of technical variation
• Corrected expression values preserve interpretable differences between conditions

## The bigger picture

Single-cell transcriptomics measures gene expression in individual cells, allowing researchers to identify different cell types and states and to examine how they change across developmental stages, diseases, and treatments. It has become an important approach for understanding biological processes at cellular resolution. As studies increasingly involve large numbers of samples and experimental batches, controlling technical variation has become essential for ensuring reliable comparisons. A major source of such variation is the batch effect, which is the technical difference introduced when samples are collected or processed separately. Because samples from different biological conditions are typically prepared in different batches, technical and biological differences between conditions can become difficult to distinguish. Most batch-correction methods remove batch effects by mapping cells from different batches into a shared latent space and making their distributions overlap. Although this approach is useful for visualization and clustering, it can also remove meaningful differences between conditions. We developed Kanade to address this limitation. Kanade is a computational method that separates information associated with batch effects, biological conditions, and cell identities, allowing technical variation to be selectively removed while condition-specific signals are retained. It also produces corrected gene-expression values that can be used for downstream analyses, including differential expression and RNA velocity.

## Graphical abstract

**Figure:**
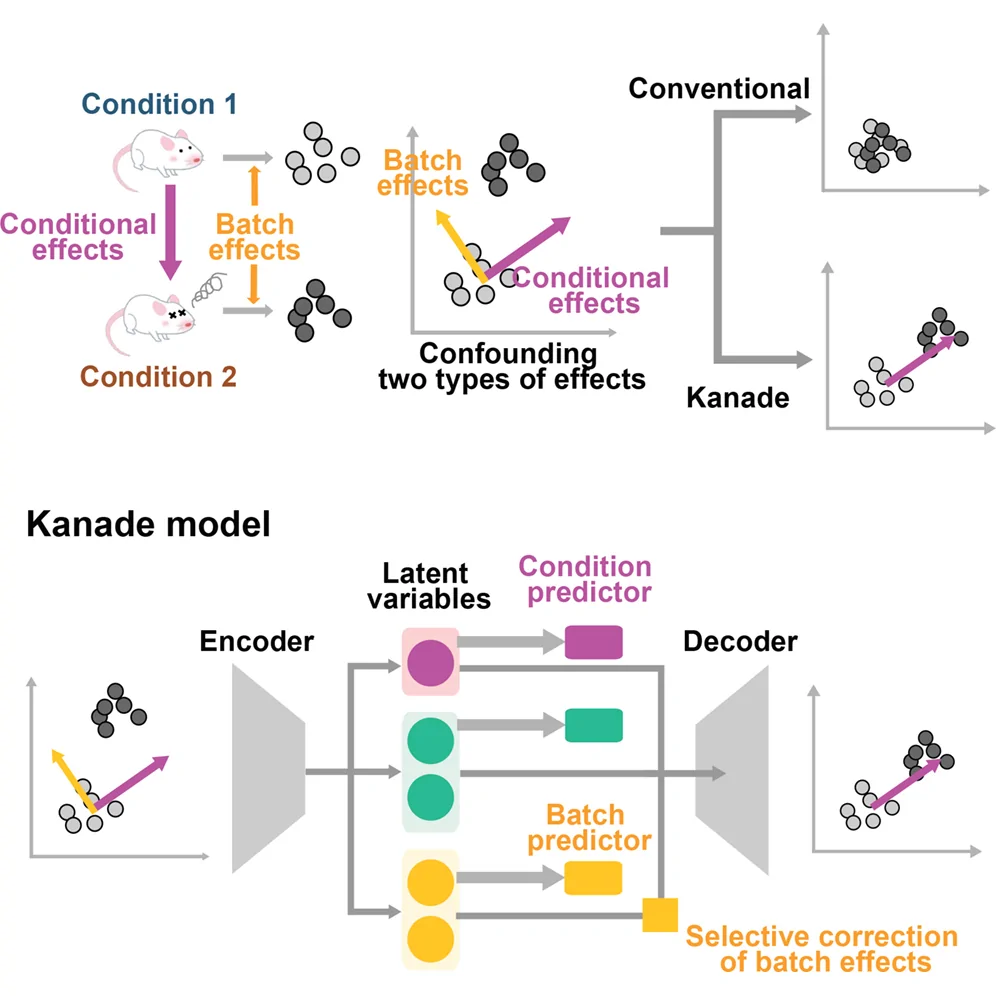

## Teaser

Batch effects can obscure biological differences in single-cell transcriptomic studies, particularly when experimental batches are confounded with disease, treatment, or developmental stages. Sakaguchi et al. introduce Kanade, a variational autoencoder that separates batch effects from condition-associated signals. Kanade selectively removes batch effects while preserving biologically meaningful differences between conditions and produces corrected gene-expression values suitable for downstream analyses, thereby enabling more reliable comparisons across complex single-cell datasets.

## Introduction

Single-cell omics is an essential technology for investigating the mechanisms of various biological processes, including cell differentiation^1^ and diseases.^2^^,^^3^ Recently, a large number of single-cell transcriptomic datasets have been obtained,^4^^,^^5^ and most of these datasets consist of multiple batches. Among the batches, unwanted variations (batch effects) arise due to differences in technical platform, experimenter, or other factors and often confound biological signals.^6^ This is an obstacle to analyzing the single-cell transcriptome dataset.

To overcome the problem, many correction methods for the batch effect have been developed. For example, Seurat^7^^,^^8^ has a data integration tool, and Harmony^9^ shows high performance in correcting batch effects.^10^ In addition, variational autoencoder (VAE)-based methods including scVI^11^ are often used. However, these methods unintentionally often remove biological signals between different conditions (e.g., health and disease), which should be preserved (Figure 1A, center-right), together with batch effects. This occurs because different conditions are often independently sampled and processed as separate batches, causing condition and batch effects to become confounded. Since comparisons between multiple conditions are fundamental to scientific research, this issue severely limits single-cell transcriptome analyses.

**Figure 1:**
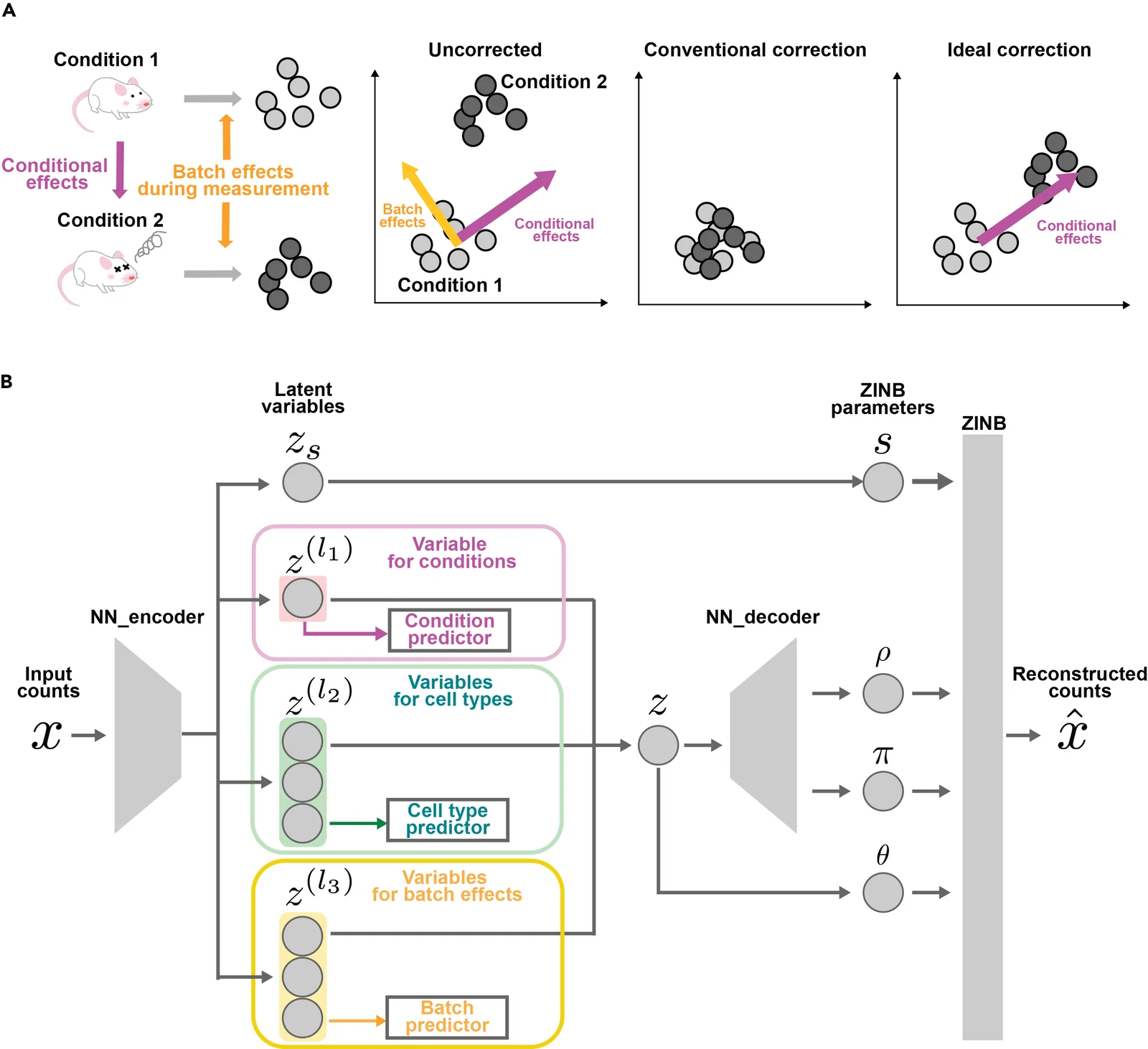
Overview of Kanade (A) A schematic illustration of the confounding of conditional effects and batch effects. Each sample obtained from different conditions is confounded by batch effects (left). The observed difference reflects the combined effects of both factors (center-left). Conventional correction methods remove both the conditional effect and the batch effect (center-right). Ideally, only the batch effect should be selectively removed while preserving the conditional effect (right). Illustrations of mice in the figure were adapted from materials by Irasutoya. (B) Schematic diagram of Kanade model structure.

A common issue with existing methods is that they do not explicitly separate confounding batch effects from biological signals. This property constrains batch-effect correction methods to strategies that seek to mix batches in the latent space, a limitation that prevents them from preserving condition-specific signals and reconstructing gene-wise expression variability. Although methods for comparing conditions, such as multiGroupVI,^12^ have been proposed in recent years, they map data to separate latent-variable spaces for each condition, making direct comparisons impossible. Consequently, most of the existing methods are used only for embedding and clustering analysis, and uncorrected counts are often directly used for other downstream statistical or biological analyses, including differential gene expression analysis or RNA velocity.^13^

One solution for the issue is to constrain sets of latent variables to assign specific roles to them. This assignment has been used in the extraction of morphological information from images. For instance, Morpho-VAE^14^ controls the latent space of the VAE to preserve morphological information for species classification by simultaneously training predictors that infer species from the latent variables. Also in single-cell omics, this approach has been introduced as SCDC^15^; however, this method focuses on separating cell-type-dependent signals from batch effects and does not explicitly account for confounding with condition-dependent signals.

Here, we propose a batch correction method called Key Approach for Noise Adjustment and DisEntanglement (Kanade; Figure 1B). The most distinctive feature of this tool is that it is designed to disentangle batch effects in transcriptomic data from biological signals between different conditions. Kanade is based on a variational autoencoder and uses predictors to assign distinct roles, such as batch-, condition-, and cell-type-related information, to separate subsets of latent variables. This makes it possible to separate batch effects from biological signals and to correct batch effects by replacing only the latent variables for batch effects with zeros or other uniform values. First, we demonstrated that Kanade was able to selectively correct batch effects and conserve conditional features using simulated datasets. Next, Kanade was applied to the single-cell RNA sequencing (scRNA-seq) atlas of pancreatic cells and corrected batch effects without losing diabetes-related features. In addition, we showed that the corrected counts by Kanade can be used for RNA velocity calculation.

## Results

### The Kanade model

Kanade is a VAE-based model (Figure 1B). Conventional VAE employs a neural network encoder to project input data into a latent space and a decoder to reconstruct the data from the latent variables. By training the neural networks to minimize the discrepancy between the reconstructed and original data, VAEs compress the information contained in the data into the latent space. However, due to the high flexibility of neural networks, it becomes unclear which features of the input are encoded in which latent variables and to what extent. As a consequence, in the context of batch correction, batch effects and condition-specific biological signals tend to be entangled in the latent space, making selective correction difficult. To address this issue, Kanade uses label predictors to guide what information is encoded in the latent variables, thereby organizing the flow of information and enabling disentanglement of batch effects from condition-dependent signals.

In the Kanade model, the expression *x*_*i*,*g*_ of gene *g* in cell *i* is assumed to be sampled from a zero-inflated negative binomial distribution (ZINB). ZINB is parameterized by the scale factor *s*, the normalized mean *ρ*, the dispersion parameter *θ*, and the zero-inflated rate *π*. Kanade uses a neural network to encode *x*_*i*_, a gene expression vector in cell *i*, into a latent variable vector *z*_*i*_ and a latent variable for scale factor $z_{s_{i}}$. $z_{s_{i}}$ is converted into the scale factor *s*, and *z*_*i*_ is decoded into other ZINB parameters. To disentangle batch effects from biological signals across different conditions, Kanade employs specific predictors. The latent variable space is partitioned into distinct sets of variables: one set is designated to predict batch labels for individual cells, while several other sets are assigned to predict the respective condition labels. The remaining set is dedicated to predicting cell types. Then, the entire model is trained so that the likelihood of ZINB for the input gene expression vector and predictability of each predictor are jointly maximized. This makes each latent variable retain corresponding information to the predictor to which it is assigned.

Specification of latent variables enables the correction of batch effects. First, data are projected into latent variables, and batch effects and signals depending on condition are stored in the corresponding set of latent variables. Then, replacing only latent variables for batch effects by zeros and reconstructing gene expressions result in corrected gene expressions.

### Biological signals and batch effects were disentangled in latent space on the simulated datasets

To evaluate Kanade, we generated a simulated dataset (methods) and applied Kanade to it. The dataset has discrete wild-type (WT)/Disease conditions (Discrete data; Figure 2A). The dataset included three cell types. Batch effects were randomly introduced in each batch (Figures 2A–2C).

**Figure 2:**
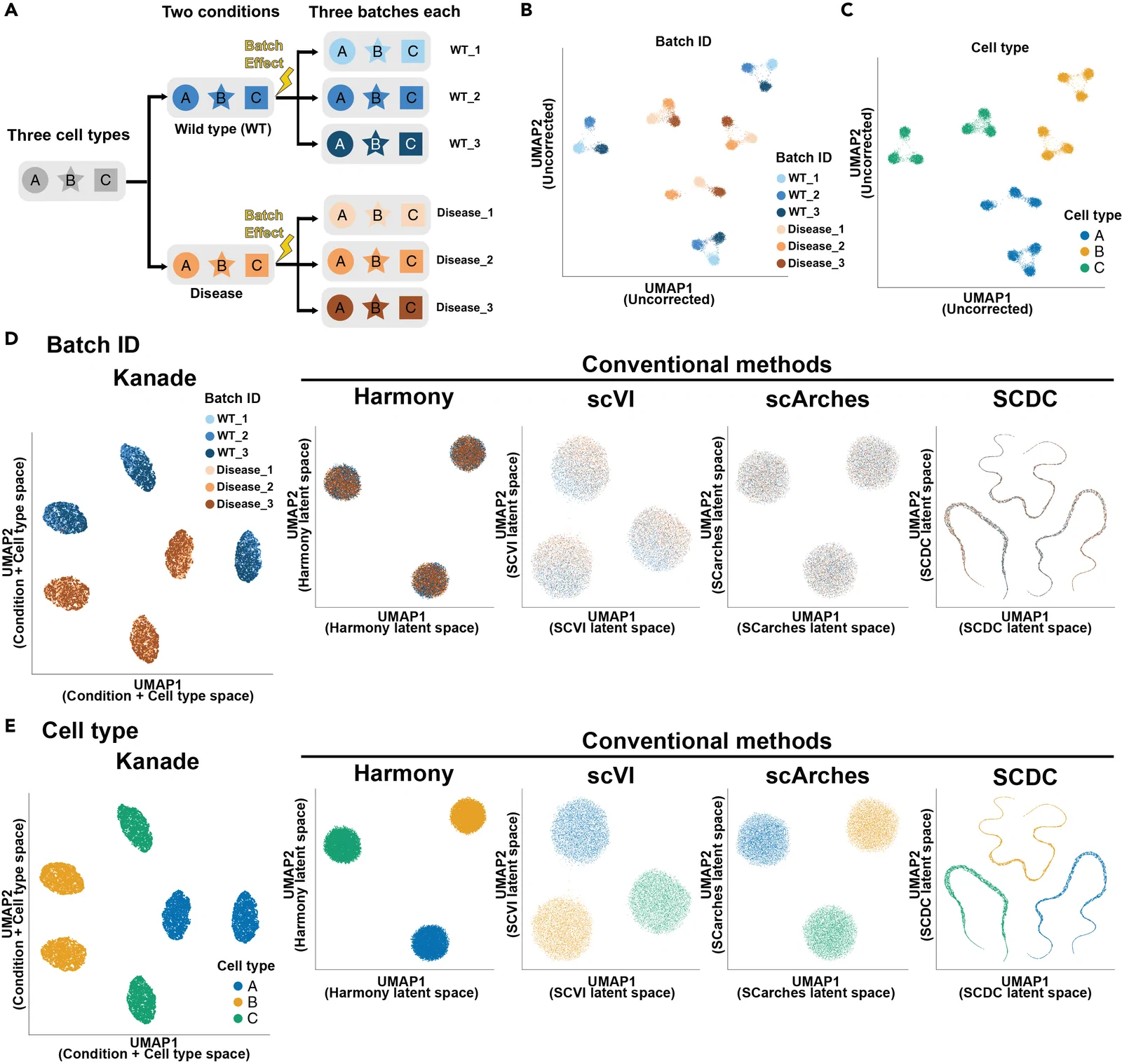
Simulated discrete data in the latent variable spaces of Kanade (A) The scheme of discrete data. (B and C) UMAP plots of discrete data based on log-normalization. Colors of dots show Batch IDs (B) and cell types (C). (D) Batch ID shown on the UMAP plots of discrete data using joint latent variables for WT/Disease condition and cell type of Kanade (left) and conventional methods (from left, Harmony, scVI, scArches, and SCDC). (E) Cell types shown on the UMAP plots of discrete data using joint latent variables for WT/Disease condition and cell type of Kanade (left) and conventional methods (from left, Harmony, scVI, scArches, and SCDC).

First, using Discrete data, we evaluated whether Kanade can separate biological signals and batch effects in the latent variable spaces. The latent variables for the WT/Disease condition showed clear differences between the two conditions, whereas they showed minimal variation among batches within the same condition (Figure S1A). Moreover, three cell types were clearly clustered in the latent variable space for cell types, and all batches were mixed in each cluster (Figures S1B and S1C). Furthermore, in the latent variable space for batches, all batches were clearly distinguished (Figure S1D). These results indicate that the three types of information were distinctly disentangled within the latent variable space. Moreover, in the joint latent space combining latent variables for the WT/Disease condition and for cell type, cells were separated according to both cell type and condition, while batches within each condition were well mixed (Figures 2D and 2E, left). We further tested Harmony,^9^ which has high performance of the batch correction^10^ and three VAE-based approaches (scVI,^11^ scArches,^16^ and SCDC^15^) on this task, all of which nearly completely mixed all batches and failed to yield distinct separation between conditions (Figures 2D and 2E, right four panels). For scVI, we tested multiple hyperparameter settings (Figure S2) and obtained largely consistent results. We also evaluated an alternative strategy in which scVI was applied separately to cells from each condition, followed by merging of the resulting latent embeddings. This strategy did not clearly separate either cell types or conditions, presumably because the embeddings were learned in independent latent spaces (Figure S3).

To evaluate these separations by Kanade quantitatively, we calculated local inverse Simpson’s indices (LISIs)^9^ for Batch IDs (Figure S4A) in each latent variable space. Inverse Simpson’s index represents the effective number of mixed components within a population, and LISI extends this concept by computing it at the single-cell level and represents the effective number of mixed components around a cell. In the absence of batch effects, where each condition contains three batches, LISI values approached 3.0 for most of the cells in the principal-component analysis (PCA) space (PCA_woBE). This means that three batches in each condition were mixed around most cells. When batch effects were introduced, the cells were segregated by batch, resulting in LISI values of approximately 1 for nearly all cells (PCA_LogNorm). This means that cells were separated into clusters consisting of single batches. In the latent variable for Disease by Kanade, LISI values were around 3.0, indicating three batches of each condition were almost perfectly mixed (z_Disease). LISIs in the latent variables for cell types (z_cell type) by Kanade showed values comparable (median 4.14) to those in Harmony-corrected variables (median 4.11), indicating a comparable level of batch effect correction to that achieved by Harmony. Although LISI values substantially exceeding 3 in the latent variables for cell types indicated over-correction as also observed in Harmony and conventional VAE-based methods, in the joint latent space of condition and cell-type latent variables (z_Disease+Cell type), LISI values were close to 3, indicating that mixing across conditions was avoided while batches were effectively mixed within each condition.

LISIs for WT/Disease conditions were also calculated (Figure S4B). LISIs for WT/Disease were close to 1 in z_Disease, as well as in PCA_woBE and PCA_LogNorm, indicating a clear separation of two conditions. In contrast, LISIs for WT/Disease in z_cell type reached almost 2.0, similar to the values observed with Harmony. These results quantitatively demonstrated the ability of Kanade to remove batch effects while preserving condition-specific information in the latent variable space. Additionally, we calculated LISIs for cell type (Figure S4C). In the latent spaces other than z_Disease, most cells showed LISI values close to 1, indicating nearly complete separation of cell types. Conversely, in z_Disease, many cells showed LISI values close to 3, suggesting that the three cell types were well mixed and that cell-type information was largely absent from this latent space.

As another evaluation, we examined whether condition, cell-type, and batch IDs could be predicted from each latent space (Table S4). First, each latent space predicted its corresponding label with near-perfect accuracy. The accuracies for predicting cell type and batch ID from the condition-specific latent space were approximately one-third, indicating nearly random performance. Similarly, the accuracy for predicting condition from the cell-type-specific latent space was approximately one-half, which was also close to random. Prediction of batch ID from the cell-type-specific latent space was also unsuccessful, with an accuracy of 0.215, although this was slightly higher than random chance (1/6). By contrast, condition could be predicted almost perfectly from the batch-specific latent space. This result reflects that once the batch is determined, the condition is also predictable and that condition information is important for predicting batch labels. Thus, complete separation between batch and condition information is not possible in this setting.

To strengthen the validation, we constructed a dataset based on the assumption of continuous time-dependent gene expression changes and cell sampling at five time points (Continuous data, Figures S5A–S5C). When Kanade was applied to the dataset, cells were separated in each latent variable space based on the corresponding signal (Figures S5D–S5G). LISI values for Batch ID in latent variables for time point (z_TP in Figure S5H) were nearly 3, indicating that batches in each time point were mixed. Similarly, LISI values in latent variables for cell type (z_Cell type in Figure S5H) showed comparable values to those in Harmony-corrected latent variables. LISIs for time points in z_TP of most cells were nearly 1, indicating separation depending on time, and those in z_Cell type showed comparable values to Harmony (Figure S5I).

### Batch effects were corrected in reconstructed counts

To test whether Kanade is able to remove batch effects in reconstructed counts and keep biological signals, the latent variables for batch effects in Discrete data were replaced by zeros, and counts were reconstructed using these corrected latent variables under the uniform scale factors. First, mean reconstructed counts of genes were highly correlated with the ground truth (Figures 3A and S6). This suggested that the expression profiles of most genes were accurately recovered. Next, dimensionality reduction was performed on reconstructed counts. Cells were clustered depending on cell types and WT/Disease conditions on the uniform manifold approximation and projection (UMAP) plot of reconstructed counts without batch-specific segregation within conditions (Figures 3B and 3C). LISIs were calculated in PCA space based on the reconstructed counts (PCA_reconst in Figure S4). The LISIs for Batch IDs exhibited a distribution similar to that observed in the absence of batch effects, while the LISIs for WT/Disease conditions were close to 1 in most cases. These results indicated that batches were mixed in the same conditions, and differences between conditions were preserved in reconstructed counts.

**Figure 3:**
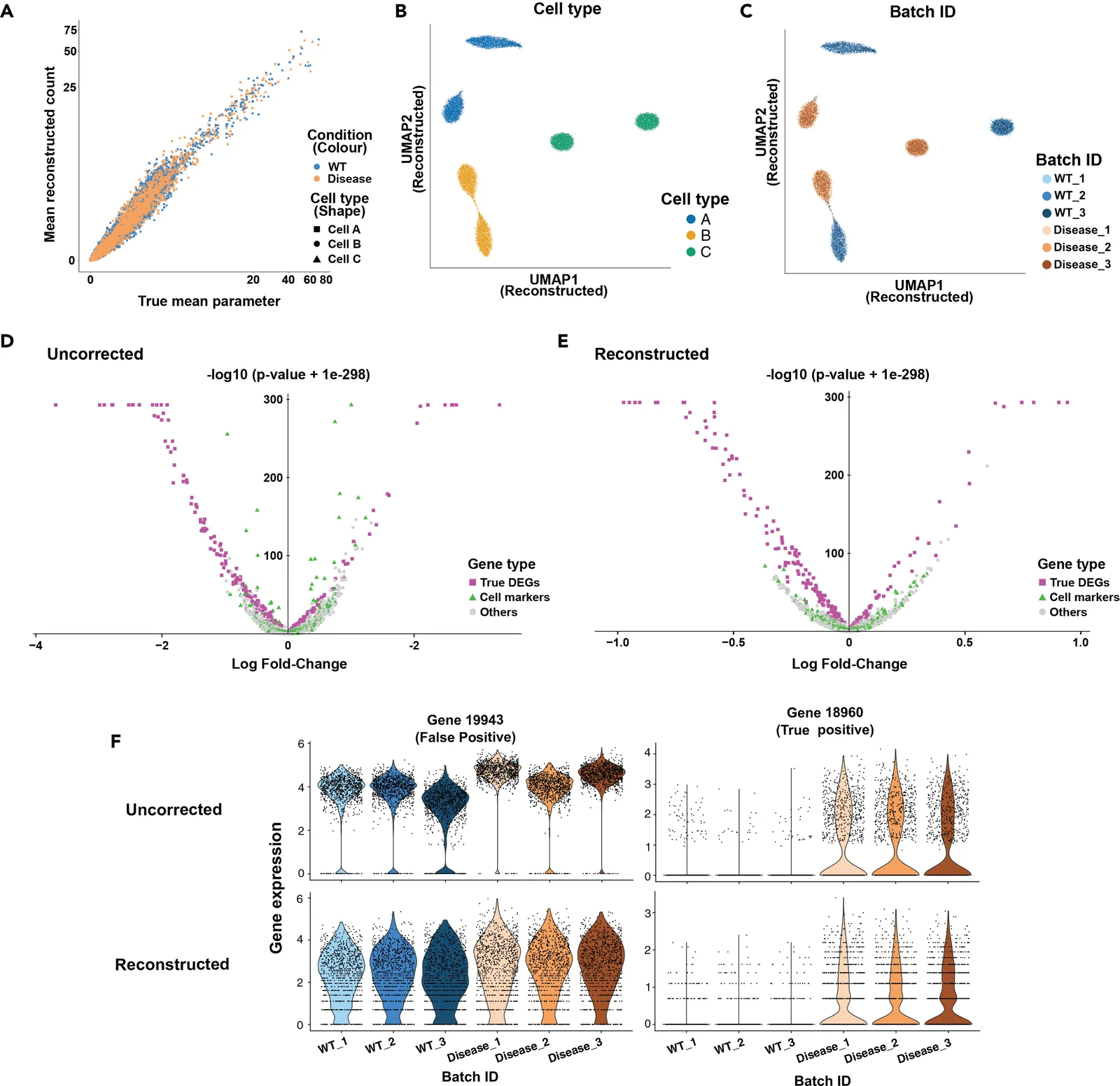
Reconstructed counts of simulated discrete data (A) Scatterplots of mean gene expressions in each cell type and condition. The *x* -axis represents the mean parameters of the simulation and the *y* axis mean of reconstructed counts. Both axes are log-scaled. Each dot represents each gene, and the color and shape of the dots represent conditions and cell types, respectively. (B and C) UMAP plots of discrete data based on reconstructed counts. The colors of the dots show cell types (B) and batch IDs (C). (D and E) Volcano plots of differentially expressed gene analysis in cell type A between WT and Disease conditions results based on uncorrected log-normalization (D) and reconstructed counts by Kanade (E). Each dot means a gene, and *x* axis represents log2-fold changes and *y* axis negative log10 *p* values. Small values (1e−293) are added to *p* values to avoid the underflow. (F) Violin plot examples of expression of false positive (left, marker gene of cell type A) genes and true positive (right) genes. Top panels show uncorrected log-normalization and bottom Kanade-corrected counts, respectively.

To verify that Kanade reconstructed meaningful counts, expressions of cell-type markers and condition-dependent genes were visualized in Figure S7. In the reconstructed counts, the markers of each cell were expressed in the corresponding cells, and the condition-dependent genes exhibited variations specifically within the corresponding cells. This result indicated that biologically meaningful counts were reconstructed. Next, to demonstrate that Kanade selectively corrected the batch effects, we performed differentially expressed gene (DEG) analysis between conditions on a specific cell type (cell type A) and drew two volcano plots. Using original counts, there was little separation between ground-truth DEGs and non-DEGs in the volcano plot but both were separated using reconstructed counts (Figures 3D and 3E). Moreover, using original counts, in spite of small log-fold changes, smaller *p* values than true DEGs were detected when cell-specific marker genes (green triangles in Figure 3D) were affected by batch effects. In contrast, using reconstructed counts, this effect was mitigated, and marker genes showed higher *p* values than true DEGs (Figure 3E). To further characterize this effect, we plotted the expression of false-positive genes (marker genes for cell-type A) and true-positive genes across batches and compared their patterns before and after correction (Figures 3F, S8, and S9). For the false-positive genes, expression levels varied substantially across batches before correction, suggesting that batch-dependent variation was misinterpreted as a significant difference between conditions. After correction, their expression became more evenly distributed across batches, making the spurious difference between conditions less apparent. In contrast, for the true-positive genes, the condition-dependent differences in expression were preserved after correction. These results indicated that Kanade distinguished batch effects from condition-dependent variations and corrected them selectively.

When Kanade was applied to Continuous data, mean reconstructed counts of genes in each cell type and time point were correlated to ground truth in the simulation (Figures S10A and S11). When dimensionality reduction on reconstructed counts was performed, cells were ordered along the time series, whereas batches in each time point were mixed (Figures S10B and S10C). LISIs for both batch IDs and time points in reconstructed counts (PCA_reconst in Figures S5H and S5I) showed similar distributions to those in counts without batch effects (PCA_woBE in Figures S5H and S5I). Moreover, in reconstructed counts, cell-type-specific marker genes were expressed specifically in corresponding cell types, and time-dependently regulated genes showed gradual variations over time (Figure S10D). In addition, as well as Discrete data, the effect in which DEG analysis calculated small *p* values of cell-type-specific marker genes by using uncorrected counts was suppressed when using reconstructed counts (Figures S10E and S10F). These results demonstrated that reconstruction by Kanade was performed well also on the Continuous data.

### Application of Kanade for mouse pancreatic datasets

To verify the performance of Kanade with real data, we applied Kanade to the scRNA-seq atlas of the Mouse pancreas (Figure 4A).^17^^,^^18^^,^^19^^,^^20^^,^^21^^,^^22^ This atlas consists of multiple datasets and includes pancreatic cells from control mice, type I (NOD) and type II (db/db) diabetes model mice, and chemical-stressed mice. Cells from db/db model mice with some treatment, vertical sleeve gastrectomy (VSG), and pair-fed (PF) were also included. The ages of mice in this atlas are not uniform. Because the ages of mice in this atlas were highly confounded with the original dataset—a major source of the batch effect—when we applied Kanade to the atlas, a latent variable to predict the age of mice was prepared in addition to a set of latent variables to predict conditions. This variable was averaged when batch correction was performed. To explore the raw data structure, we generated the UMAP plot based on uncorrected counts and found some clusters were separated depending on batches or original datasets in spite of the same conditions and cell types (Figures 4B and 4C). As the input cell types, we roughly cluster cells using Harmony, ignoring conditions (Figures S12A and S12B; see methods).

**Figure 4:**
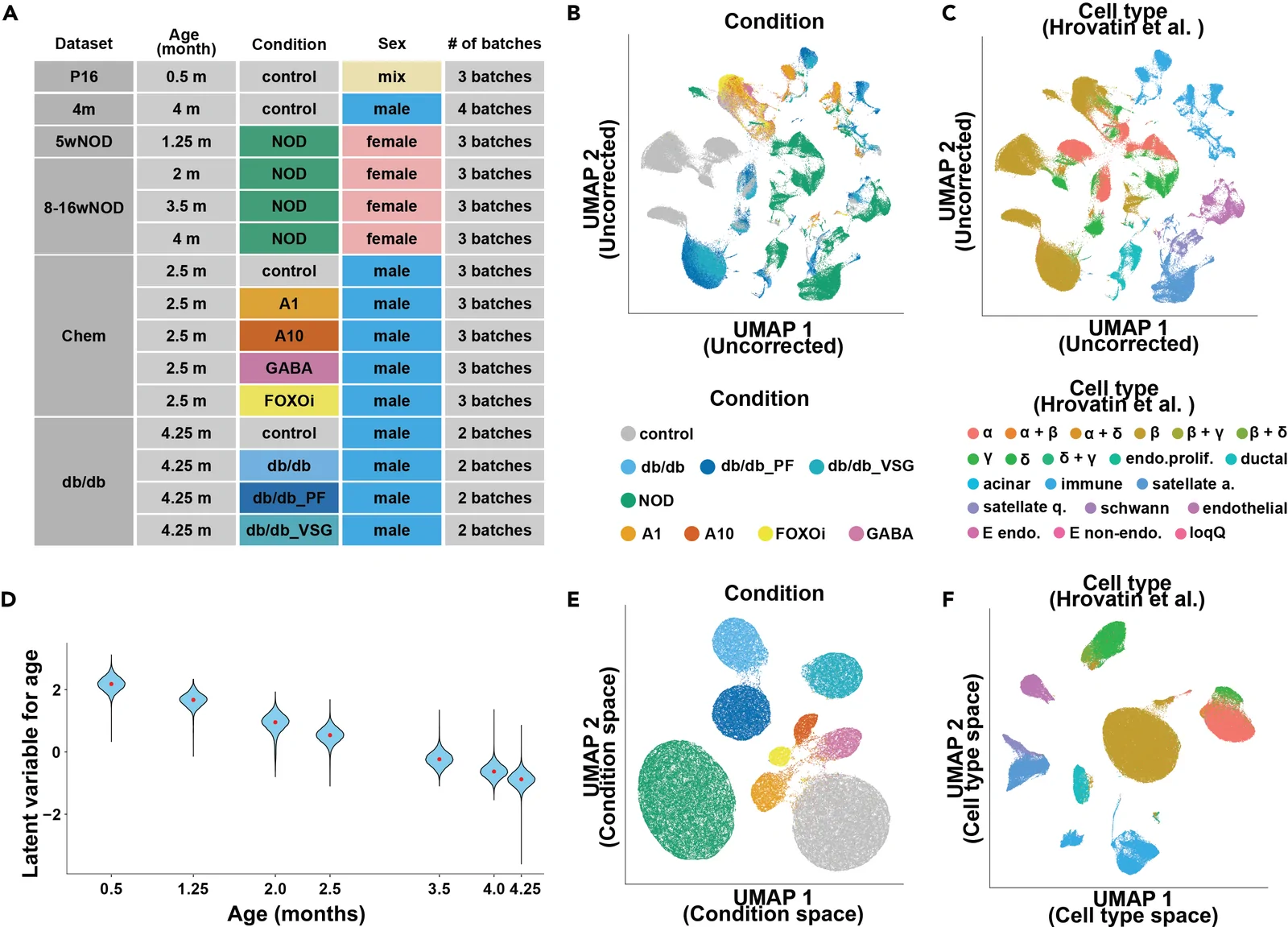
Dimensionality reduction of mouse pancreatic atlas (A) Overview of the pancreatic atlas used in this study from Hrovatin et al. (B and C) UMAP plots of the pancreatic atlas based on log-normalized counts. The colors of dots show the condition (B) and cell type annotated by Hrovatin et al. (C) of the cells. (D) Violin plot showing the distribution of the latent variable for age at each time point. The *x* axis represents age in months, with each violin positioned accordingly. Red dots indicate the median values at each time point. (E) UMAP plot of the pancreatic atlas based on latent variables for conditions. The colors of the dots show the conditions of the of cells. Colors are the same as (B). (F) UMAP plot of the pancreatic atlas based on latent variables for cell types. The colors of the dots show the cell type annotated by Hrovatin et al. of cells. Colors are the same as (C) A1 and A10: artemether (1 or 10 μM); FOXOi: FoxO inhibitor endo.: endocrine; prolif.: proliferating; stellate a.: stellate-activated; stellate q.: stellate-quiescent; low-Q: low quality cell; E: embryonic (misannotated). *α* + *β*, *α* + *δ*, *β* + *γ*, *β* + *δ*, and *δ* + *γ* means doublet of two cell types.

First, we found the encoder of Kanade was able to separate the signals in the latent variable space. The medians of latent variables for ages were clearly correlated with ages of input labels, whereas batches from the same time points showed small differences among them (Figures 4D and S13). In the latent variable space for conditions, cells were separated depending on the conditions, and batches in the same conditions were mixed (Figures 4E and S14). Importantly, control cells from different datasets were mapped into the same clusters (Figure S14A). In the latent variable space for cell types, cells were separated by the input labels but not batches (Figures S12C and S15). Interestingly, we found more detailed cell types than input labels were distinguishable in the space (Figures 4F and S16A). For example, alpha cells and gamma cells were separated in the UMAP plot in spite of both being labeled as input cluster 1. Moreover, lymphoid cells (input cluster 3) were separated into B cells, plasma cells, T cells, and the cell-cycling population of each cell type in the UMAP plot (Figure S16A). These results suggest that Kanade is robust for a coarse input about cell types, and fine-grained annotations are not necessary.

Next, we validated whether reconstructed counts contained biological features. In the UMAP plot based on the reconstructed counts, cells were clustered based on cell types and conditions, and no batch-specific clustering was observed (Figures 5A–5C and S17). Note that control cells were mapped into the same clusters regardless of the source dataset as well as the latent variable space (Figure S17). This suggests that batch effects were corrected well. Each cell type expressed the corresponding marker gene, suggesting the reconstructed counts retained distinctive cell-type features (Figure S18D). It is important that, as in the case of the latent variable space, more detailed cell types than input labels were distinguishable in the reconstructed counts. For example, potential doublet cells of pancreatic alpha cells and beta cells co-expressed glucagon (*Gcg*) and insulin I (*Ins1*). In addition, lymphoid cells (B cells, T cells, and plasma cells) showed cell-type-specific gene expression patterns.^23^^,^^24^^,^^25^^,^^26^^,^^27^^,^^28^^,^^29^

**Figure 5:**
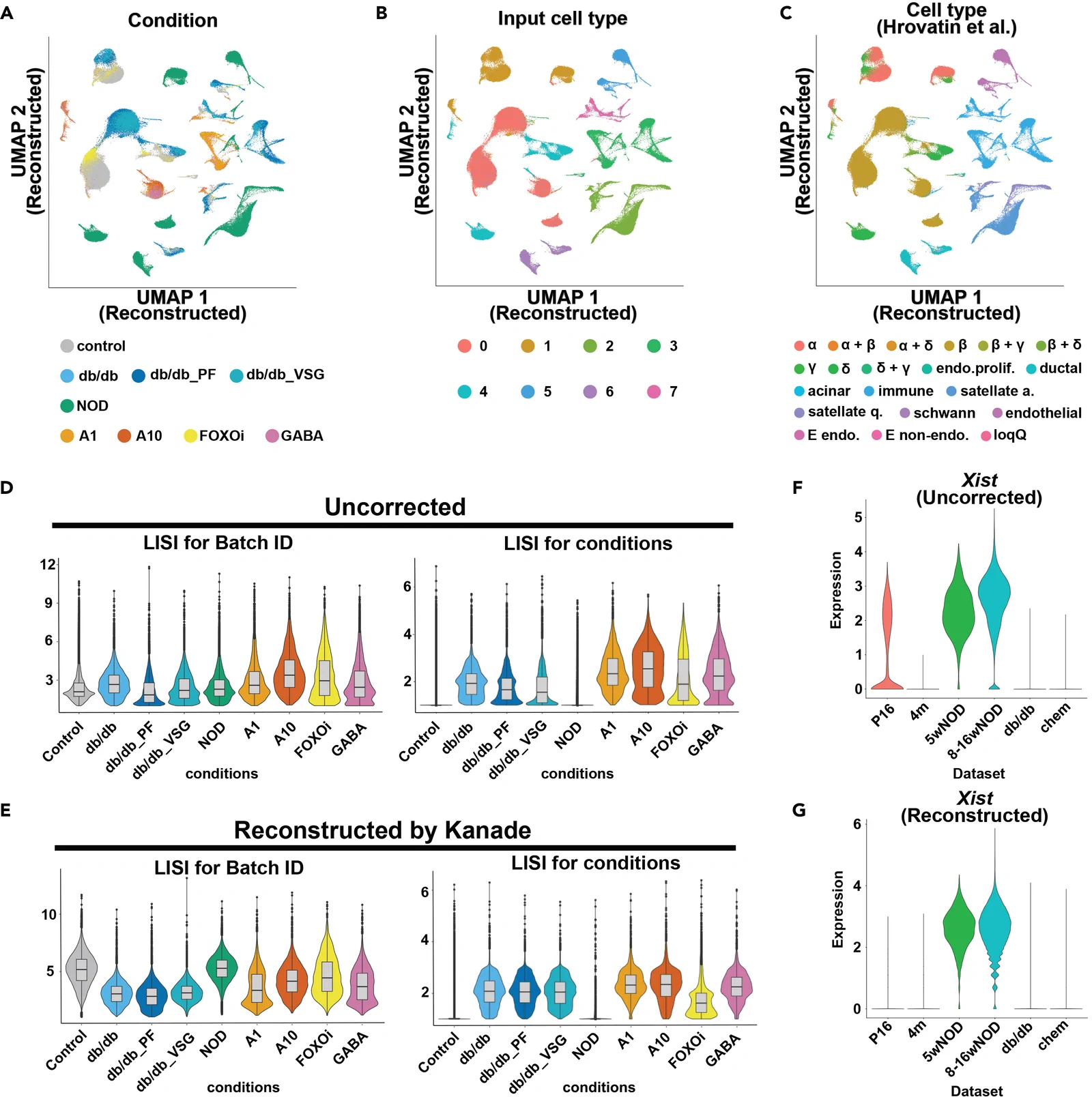
Reconstructed counts of the mouse pancreatic atlas (A–C) UMAP plots of pancreatic atlas based on reconstructed counts. The color of the dots show conditions (A), input cell types (B), and cell types annotated by Hrovatin et al. (C). (D and E) Violin plots of LISI (left) for Batch ID and (right) for conditions, respectively. LISIs are based on (D) uncorrected counts and (E) Kanade-corrected counts. Cells are grouped by conditions. (F and G) Violin plots of expression of *Xist* in each dataset based on uncorrected counts (F) and reconstructed counts (G). A1 and A10: artemether (1 or 10 μM); FOXOi: FoxO inhibitor endo.: endocrine; prolif.: proliferating; stellate a.: stellate-activated; stellate q.: stellate-quiescent; low-Q: low quality cell; E: embryonic (misannotated). *α* + *β*, *α* + *δ*, *β* + *γ*, *β* + *δ*, and *δ* + *γ* means doublet of two cell types.

To provide a more quantitative evaluation, we calculated LISI in the PCA space after correction and visualized the values for each condition using violin plots. We then compared these results with those obtained before correction and with the latent spaces generated by other VAE-based methods (Figures 5D, 5E, and S19). Before correction, many cells showed an LISI for batch ID close to 1, whereas such cells were rarely observed after correction, indicating effective removal of batch effects. In addition, the LISI for condition remained below 4 for many cells after correction. This pattern was distinct from those observed for Harmony, scArches, and SCDC, which showed long tails toward higher values, suggesting excessive mixing between conditions. Compared with scVI, Kanade showed a similar distribution of LISI for condition; however, scVI retained many cells with an LISI for batch ID close to 1 in several conditions, such as db/db_PF and FOXOi, indicating insufficient correction of batch effects. UMAP plots of the spaces used for comparison are shown in Figure S20.

To demonstrate whether Kanade was able to selectively correct batch effects and keep conditional features, we focused on the expression of inactive X-specific transcripts (*Xist*). This gene is the one expressed only in females.^30^ In the atlas used in this study, most cells are of male origin, while all cells in the NOD condition and only some of the control cells from the P16 dataset are of female origin (Figure 4A). Consistent with this, *Xist* was detected in all batches under the NOD condition and only in batches derived from P16 under the control condition but hardly detected in other batches under the control condition or any batches under other conditions before the correction (Figure 5F). In this context, *Xist* expression can be regarded as a pseudo-NOD-specific feature, and its expression in P16 dataset cells arose from dataset-specific differences, allowing it to be considered a batch effect. Surprisingly, in the reconstructed counts, *Xist* expression in cells from the P16 dataset almost disappeared, while it was maintained in cells from the two datasets under NOD condition (Figure 5G). This result indicated that Kanade was able to distinguish between batch effects and condition-specific features, enabling selective correction of batch effects.

Furthermore, to demonstrate the ability of Kanade to conserve conditional features in a diabetic context, we focused on pancreatic beta cells. First, expressions of diabetes-associated marker genes shown in Hrovatin et al. were examined (Figure 6A). In the NOD condition, beta-2 microglobulin (*B2m*), a type I diabetic marker,^31^ showed higher expression than other conditions, and three db/db conditions showed higher expression of type 2 diabetic markers: aldehyde dehydrogenase family 1 subfamily A3 (*Aldh1a3*),^32^ vitamin-D-binding protein (*Gc*),^33^ cholecystokinin (*Cck*),^34^ and gastrin (*Gast*).^35^ Next, PCA was performed on reconstructed counts of beta cells and principal components (PCs) 3 and 2 separated the NOD and db/db conditions, respectively (Figure 6B). GO terms related to the immune system including “innate immune response” (GO:0045087) were enriched in the top 100 genes that negatively contributed to PC 3 (Figure S21A). These results were consistent with the fact that type 1 diabetes is an autoimmune disease.^36^ GO terms related to the response to nutrition and diet including “response to food” (GO:0032094) were enriched in the top 100 genes that negatively contributed to PC 2 (Figure S21B). In addition, PC 2 and PC 6 separated db/db_VSG from db/db mice, but no PC was found that separated db/db_PF conditions (Figure S21C). In fact, expressions of *Cck* and *Gast*, genes with up-regulation in db/db conditions, were significantly downregulated in db/db VSG compared to db/db (*p* below numerical precision and *p* = 2.02 × 10^−57^, respectively; Figures S21D and S21E). These are consistent with the original paper of the dataset, which showed VSG results in a beta cell recovery from the diabetic characters but not PF.^21^ Therefore, Kanade was able to preserve the features in the diabetes-related context.

**Figure 6:**
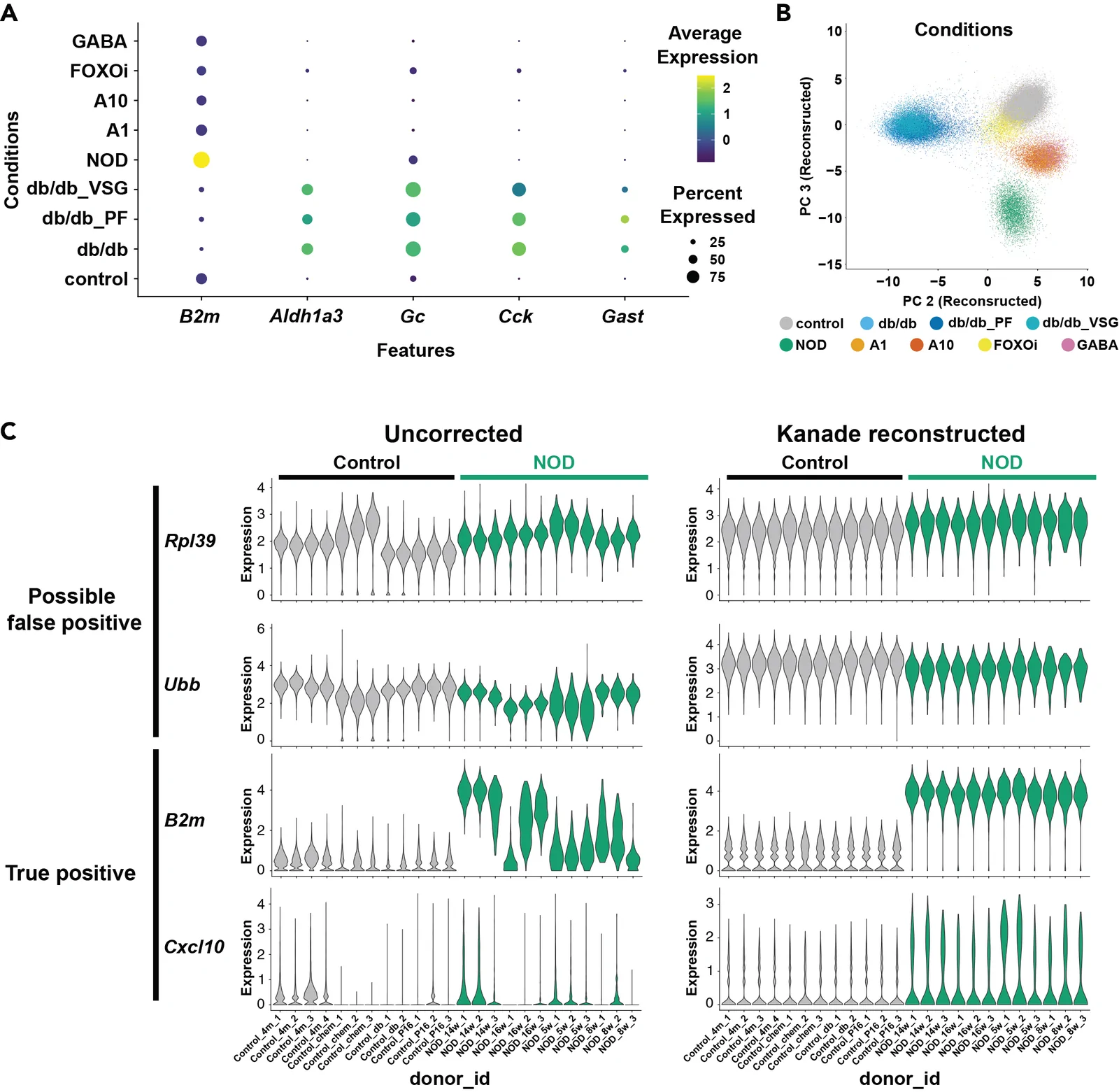
Conservation of conditional effects among conditions in the reconstructed counts of beta cells (A) Dot plot of expressions of marker genes of type 1 diabetes and type II diabetes in beta cells. (B) PCA plot of beta cells. The *x* axis and the *y* axis represent PC 2 and PC3, respectively. The colors of the dots show the conditions of cells. (C) Violin plots of expressions of *Rpl39*, *Ubb*, *B2m*, and *Cxcl10* from top, respectively. Left panels show uncorrected counts, and right panels show Kanade-corrected counts. Cells were grouped by batches. Color represents conditions. Gray: control; green: NODA1 and A10: artemether (1 or 10 μM); FOXOi: FoxO inhibitor.

To examine the effect of batch correction in more detail, we performed DEG analysis between NOD condition and control for beta cells using expression values before and after correction. In each analysis, genes were ranked by ascending *p* value. Among the genes ranked within the top 200 before correction, those showing the largest decreases in rank after correction were regarded as potential false-positive genes, and we examined how their expression patterns were altered by correction. The potential false-positive genes included ribosomal protein genes and ubiquitin, suggesting an influence of batch effects. Indeed, when the expressions of ribosomal protein L39 (*Rpl39*) and ubiquitin B (*Ubb*) were plotted using the uncorrected values, substantial fluctuations were observed across batches. After correction by Kanade, these batch-dependent differences were reduced (Figure 6C, top two panels). For comparison, we examined the expression of *B2m* and C-X-C motif chemokine ligand 10 (*Cxcl10*), which are true-positive genes whose upregulation has been experimentally validated^31^^,^^37^ (Figure 6C, bottom two panels). Before correction, their expression also varied across batches; however, after correction, variability among batches within the same condition was reduced, making the differences between conditions clearer in the violin plots. These results suggest that batch correction by Kanade improves biological interpretability in the context of comparisons between conditions.

### Application of Kanade for various datasets

To make the evaluation of Kanade more comprehensive, we applied Kanade to multiple datasets and compared its correction performance with that of other methods. First, we used the mouse and human immune cell dataset compiled by Luecken et al.^38^ to examine whether batch effects could be corrected while preserving species-specific differences between humans and mice. However, batches generated using Smart-seq2 were excluded because their total expression levels differed markedly from those of the other datasets and because they were present only in the human data.

For this analysis, the UMAP plots and LISI values for batch ID are shown in Figure 7. Before correction, strong batch effects were observed, with a large number of cells showing LISI values close to 1. After correction by Kanade, species and cell types remained well separated on the UMAP, whereas a small number of cells with LISI values close to 1 were observed. This suggests that Kanade removed batch effects while preserving interspecies differences. In this dataset, the latent spaces generated by Harmony, scVI, and SCDC also retained species-specific differences and cell-type information. However, the LISI values in these latent spaces showed that many cells still had values close to 1, suggesting incomplete removal of batch effects. scArches is designed to integrate query data into a reference; however, at least in our setting, it failed to learn the query data adequately. This should not be interpreted as a general limitation of scArches; rather, it may reflect differences between our preprocessing strategy and the workflow used in the original scArches study. Specifically, we used all genes for all methods to maintain consistency across comparisons, whereas the original scArches workflow performs correction after selecting highly variable genes.

**Figure 7:**
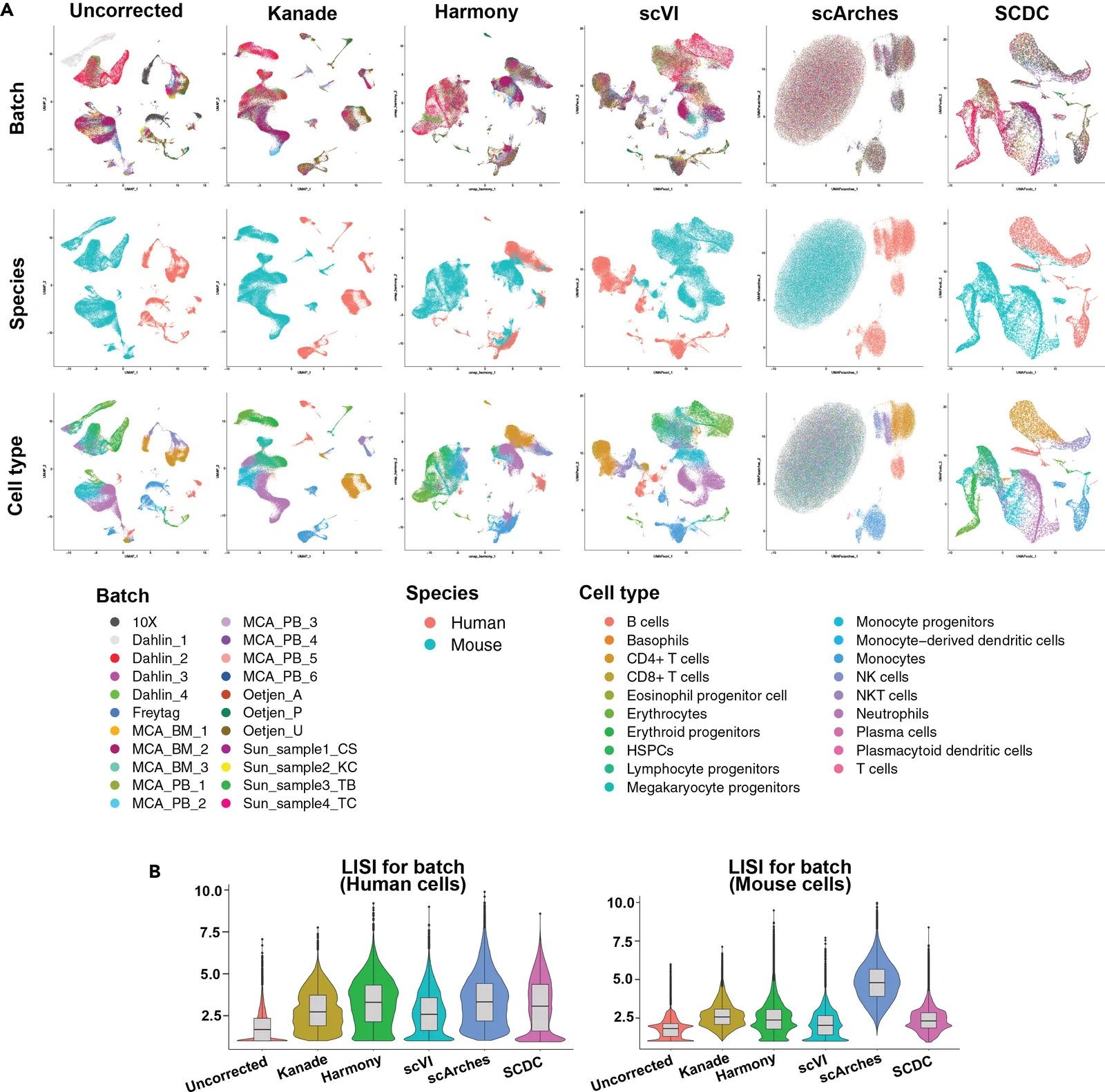
Comparison of correction results for human and mice immune dataset among methods (A) UMAP plots based on each methods; from left, uncorrected, Kanade-corrected, Harmony, scVI, scArches, and SCDC, respectively. From top, colors show Batch ID, species and cell types, respectively. (B) Violin plots of LISI for Batch ID based on each methods. Plots were separated by species: human (left) and mouse (right).

In addition, we tested Kanade using the SEA-AD dataset.^39^ This dataset consists of postmortem brain samples from cognitively normal individuals and patients with dementia. In the uncorrected data, however, both disease-associated differences and batch effects appeared to be relatively small (Figure 8, the leftmost column). Because Kanade uses both condition and batch labels during training, there was a potential concern that, in such a dataset, it might artificially amplify differences that were not clearly present in the original data. When Kanade and the other methods were applied to the SEA-AD dataset, all methods showed slight differences between normal and dementia samples, but cells from different batches were broadly integrated within each cell type (Figure 8, right four columns). These results suggest that, when applied to data with only modest differences between conditions, Kanade behaves similarly to other batch correction methods and does not exaggerate condition-associated differences. Note that scArches was excluded from this comparison because it failed to learn the reference data, presumably for the same reason as described above.

**Figure 8:**
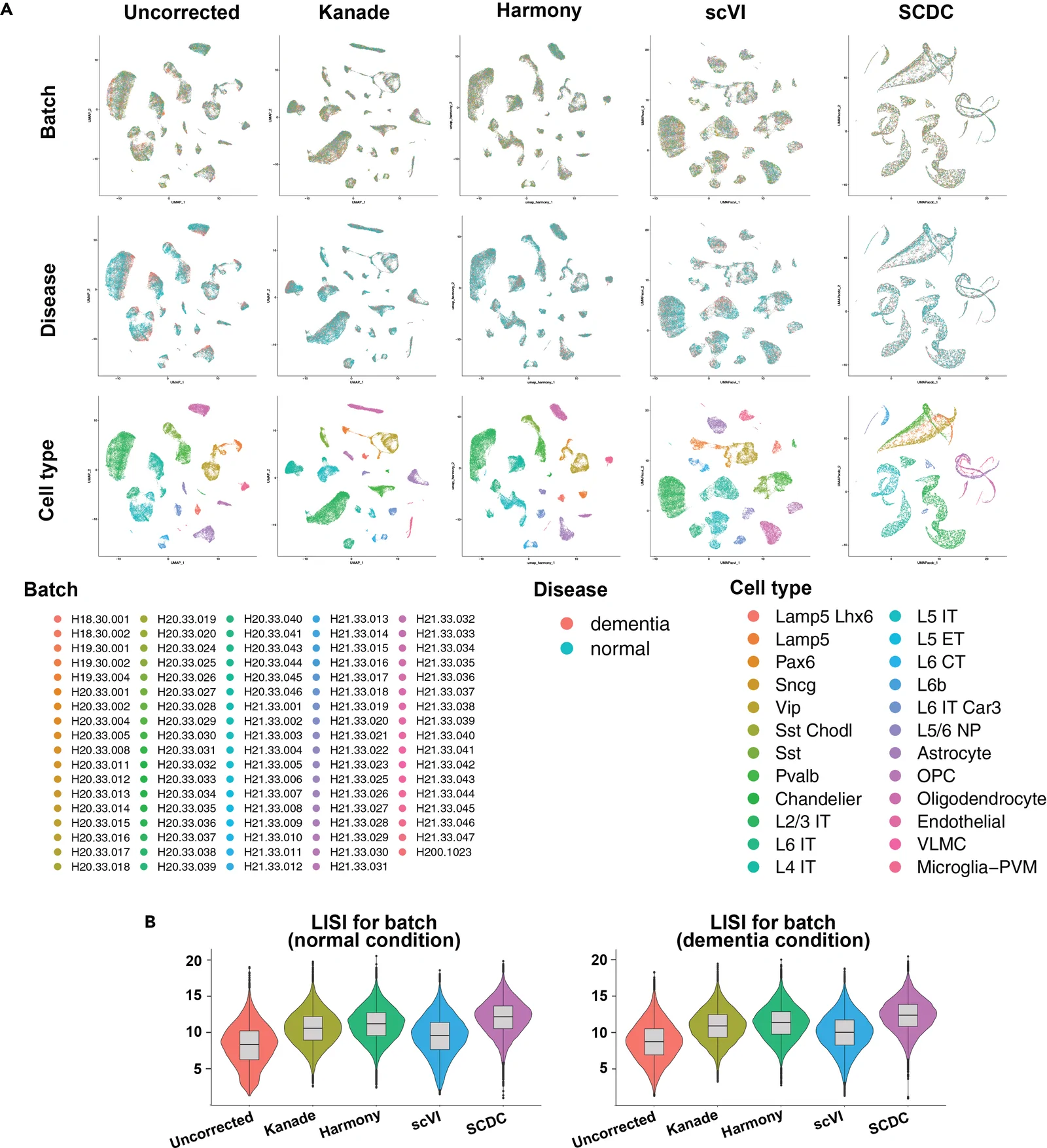
Comparison of correction results for SEA-AD dataset among methods (A) UMAP plots based on each methods; from left, uncorrected, Kanade-corrected, Harmony, scVI, and SCDC, respectively. From top, colors show Batch ID, disease condition, and cell types, respectively. (B) Violin plots of LISI for Batch ID based on each methods. Plots were separated by conditions: normal (left) and dementia (right).

Finally, Kanade is expected to have an advantage for downstream methods that rely on gene-level counts because an important feature of Kanade is that batch correction is performed on the reconstructed counts rather than on the latent variables. Then, we investigated whether the batch corrected data by Kanade were suitable for computing RNA velocity. We applied Kanade to the dataset of mouse gastrulation^40^^,^^41^ and calculated RNA velocity based on both the reconstructed and original counts using scVelo.^41^ RNA velocities calculated based on reconstructed counts showed a similar pattern as those on original counts (Figure 9). This result suggested that batch correction by Kanade preserves information on cellular dynamics.

**Figure 9:**
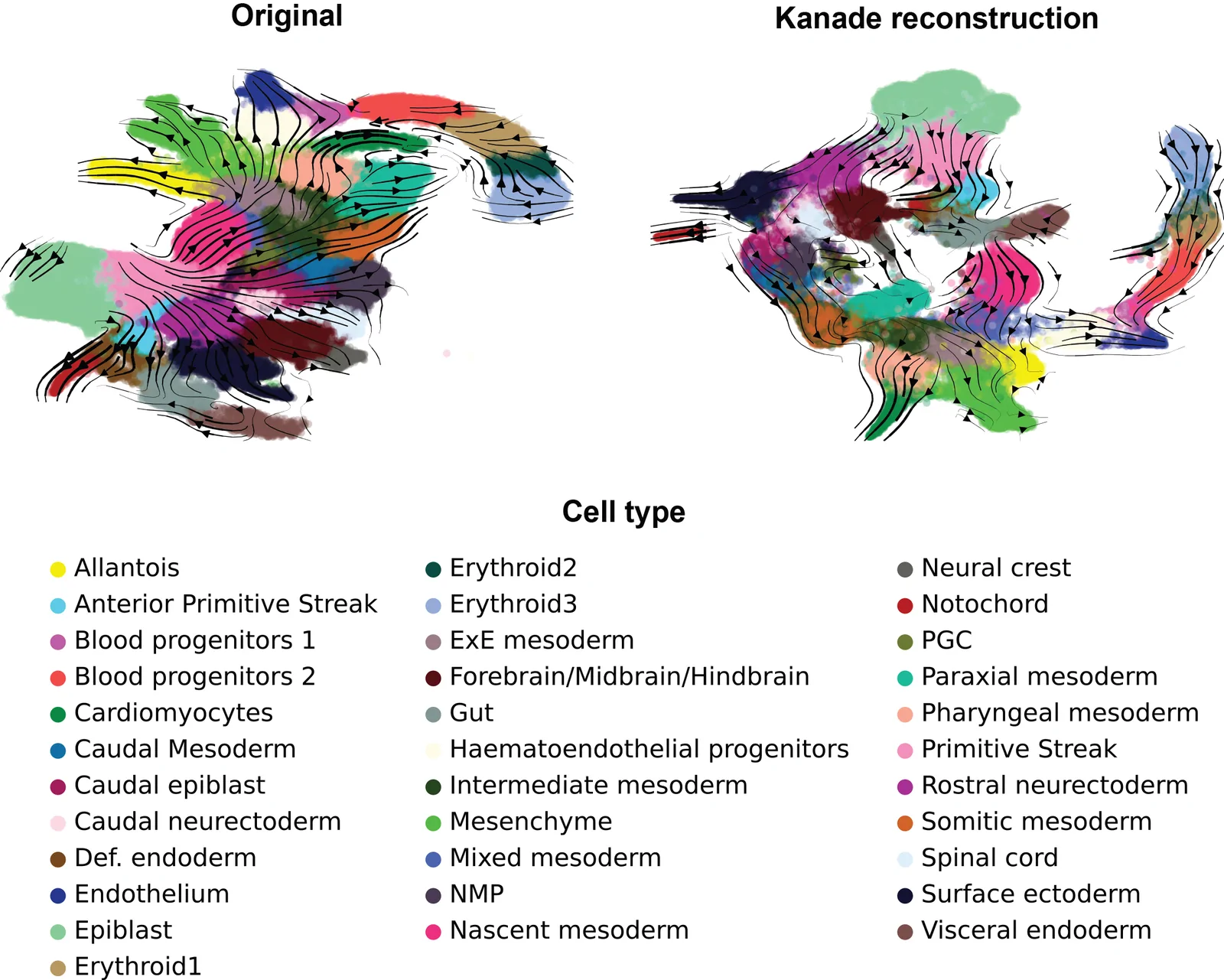
Calculation of RNA velocity based on the reconstructed counts RNA velocity of mouse gastrulation data based on the original counts (left) and the reconstructed counts by Kanade (right). The colors of the dots show the cell types.

### Evaluation of the effects of cell-type label uncertainty

Here, we examined the dependence of Kanade on input labels. During training, Kanade requires condition, cell-type, and batch labels. The method assumes an experimental setting in which multiple samples are available for each condition and are prepared and annotated separately. In such datasets, each sample, or each cell, is typically associated with condition and batch labels. In contrast, cell-type labels often need to be assigned separately for each batch or after applying a conventional batch correction method and may therefore contain annotation errors. It is therefore important to assess whether such errors affect the training of Kanade.

To address this question, we generated a modified version of the Discrete Data described above, in which 10% of the labels for cell-type A and cell-type B were swapped, corresponding to 600 of 6,000 cells (label-swapped data). When Kanade was trained on this dataset, the label-swapped cells were integrated with cells of their original cell types in both the cell-type-specific latent space and the reconstructed space (Figures S22A and S22B). In addition, examination of marker gene expression for each cell type showed no ectopic expression patterns (Figures S22C and S22D). These results suggest that, at least under this setting, the introduced cell-type label errors had only a limited effect on the resulting latent representations and reconstructed expression profiles.

To further evaluate a more realistic setting, we focused on the mouse pancreas dataset and examined cells for which the original annotation by Hrovatin et al. differed from the coarse annotation that we used for training, as these cells can be regarded as cases of inaccurate or ambiguous annotation. First, among cells assigned to cluster 0 in our annotation, which mainly corresponds to beta cells, some cells were annotated as non-beta cells in the original annotation. We focused on alpha cells among these cells and examined the expression of *Gcg*, a marker of alpha cells, within cluster 0. Cells with high *Gcg* expression before correction, many of which were annotated as alpha cells in the original annotation, retained high *Gcg* expression after correction (Figure S23A). This suggests that the effect of our annotation was limited. Conversely, we also focused on cells annotated as alpha cells in the original annotation but assigned to clusters other than cluster 1 in our annotation, where cluster 1 mainly corresponds to alpha cells. These cells also retained high *Gcg* expression after correction (Figure S23B, left). In addition, the expression levels of *Ins1*, a beta-cell marker, and *Sst*, a delta-cell marker, showed significant correlations before and after correction (*Ins1*: Spearman’s *ρ* = 0.49, *p* < 2.2 × 10^−16^; *Sst*: Spearman’s *ρ* = 0.60, *p* < 2.2 × 10^−16^; Figure S23B, middle and right). These results suggest that Kanade is relatively insensitive to the types of cell-type annotation uncertainty examined here.

## Discussion

We developed Kanade, a VAE-based method to disentangle condition-specific features from batch effects and to selectively correct batch effects. For both simulated and real datasets, we demonstrated that Kanade separated condition-specific features and batch effects in latent variable spaces and preserved condition-specific features in the reconstructed and corrected counts. In addition, we showed that batch correction by Kanade preserved the information on cell dynamics. Comparisons between conditions are fundamental to biological research, and a correction method dedicated to this purpose has been an important need. Our method is expected to meet that need and be applied to research in medicine and drug discovery and more fundamental fields including developmental biology.

A key characteristic of Kanade is its ability to explicitly separate information by assigning predictors of specific attributes to designated subspaces in the latent variable space. As a result, batch effects can be selectively corrected without interfering with condition-specific features. Moreover, unlike existing batch correction methods including scVI and Harmony, which primarily focus on embedding data into a shared latent space, Kanade leverages the advantages of generative modeling to achieve batch correction in the reconstructed expression space. This feature enables calculation of count-based downstream analysis including RNA velocity and enhances interpretability, thereby improving accessibility for biological researchers. In addition, the combination of generative modeling and explicit disentanglement can enable potential applications such as interpolation between biological conditions or simulating intermediate states. Because Kanade separates information into modular latent components, each subspace remains interpretable and adaptable to downstream tasks.

Another important property of Kanade is its simple architecture. It is built on a standard variational autoencoder framework and basic prediction models such as the logistic regression. It does not require advanced knowledge of machine learning for users or developers to understand and modify. Thus, not only the machine learning experts but also experimental biologists are well positioned to extend Kanade depending on their interests. It is expected that integrating Kanade with various statistical or machine-learning models or biological knowledge may accelerate progress in single-cell omics research. In conclusion, Kanade offers a flexible approach to selectively correcting batch effects while preserving condition-specific information and balancing interpretability and usability in batch correction in single-cell omics.

### Limitations of the study

Kanade has certain limitations. The most important limitation is its dependence on input labels, especially cell-type labels. Cell-type labels need to be assigned independently before applying Kanade. Although our results suggest that Kanade is relatively robust to annotation errors and can tolerate moderately coarse annotations, the granularity of cell-type labeling may still affect the analysis. Second, it is fundamentally difficult to completely separate condition-dependent signals from batch effects in this framework. This is because, once the batch is known, the condition can often be inferred, and conversely, once the condition is known, the possible batch labels can be substantially narrowed down. Therefore, complete disentanglement cannot be achieved by simply using discriminative predictors. Addressing these two limitations will be an important direction for future work. Given the high extensibility of Kanade, potential solutions include refining the cost function or introducing additional constraints to promote more effective and label-independent separation.

Third, Kanade is unable to handle previously unseen batches. This limitation likely stems from the fact that the model has not been exposed to a sufficiently diverse range of batch-specific variations during training. It is possible that training on a larger and more diverse dataset may allow the model to evolve into a more robust foundation model capable of handling broader datasets. Finally, it is challenging to disentangle dataset-wide shared information, such as variations arising from differences across laboratories. This limitation could be addressed by ensuring that each laboratory sets appropriate controls when conducting experiments, thereby enabling the incorporation of a predictor for laboratory identity into Kanade and the disentanglement of laboratory effects.

## Methods

### Kanade model

Kanade assumes that *x*_*g*,*i*_, the gene expression count of gene *g* in cell *i*, is sampled from the zero-inflated negative binomial (ZINB) distribution defined by latent variables. This process can be written down as follows:

$$z_{i}∼\text{Normal}\left(0,I\right)$$

$${z_{s}}_{i}∼\text{Normal}\left(0,1\right)$$

$$s_{i}=s_{0}\;\exp\left({z_{s}}_{i}\right)$$

$$\rho_{g,i}=f_{g}^{\rho }\left(z_{i}\right)$$

$$\pi_{g,i}=f_{g}^{\pi }\left(z_{i}\right)$$

$$\theta_{i}=f^{\theta }\left(z_{i}\right)$$

$$\lambda_{g,i}∼\text{Gamma}\left(\theta_{i},\theta_{i}/s_{i}\rho_{g,i}\right)$$

$$y_{g,i}∼\text{Poisson}\left(\lambda_{g,i}\right)$$

$$h_{g,i}∼\text{Bernoulli}\left(1-\pi_{g,i}\right)$$

$$x_{g,i}=\left\{ \begin{array}{cc} y_{g,i} & \text{if}\;h_{g,i}=1\\ 0 & \text{if}\;h_{g,i}=0 \end{array}\right.$$

The prior distributions of latent variables $z_{i}\in Z^{K}$ and $z_{si}\in \mathbb{Z}$ are assumed to be independent normal distributions. The scale factor *s*_*i*_ is calculated as an exponential of $z_{si}$. From *z*_*i*_, the parameters of ZINB *ρ*_*g*,*i*_ and *π*_*g*,*i*_ are calculated by function $f_{g}^{\rho }\left(z_{i}\right)$ and $f_{g}^{\pi }\left(z_{i}\right)$, respectively. These functions are modeled by a neural network (NN_decoder), and outputs are scaled by softmax function for *ρ*_*g*,*i*_ and logistic function for *π*_*g*,*I*_, respectively. The parameter *θ*_*i*_, common in all genes, is calculated by a function $f_{g}^{\theta }\left(z_{i}\right)$, the softplus function of the weighted sum of elements of *z*_*i*_.

The parameter *λ*_*g*,*i*_ of Poisson distribution is sampled from the Gamma distribution with shape parameter *θ* and rate parameter *θ*/*s*_*i*_*ρ*_*g*,*i*_, and a true count *y*_*g*,*i*_ is sampled from Poisson distribution with the sampled *λ*_*g*,*i*_. To inflate zero-values, the count *x*_*g*,*i*_ is forced to zero with probability *π*_*g*,*i*_, otherwise *x*_*g*,*i*_ = *y*_*g*,*i*_.

Kanade modeled this process as a variational autoencoder-based model using PyTorch.^42^ The input of Kanade is *x*_*i*_, and the encoder neural network, NN_encoder, projected the input into means *μ* and variances *σ*^2^ (*σ*^2^ is log-scaled) of latent variable *z*_*i*_ and ${z_{s}}_{i}$. *z*_*i*_ and ${z_{s}}_{i}$ are sampled using the parameterization trick based on calculated mean and variance. From *z*_*i*_, ZINB parameters are calculated in the way explained above. In this paper, the NN_encoder had three layers, and the numbers of nodes were 2000, 400, and 200 from input, respectively. Each layer consisted of batch normalization, ReLU activation, and dropout sublayers in this order. NN_decoder had three layers with the same sublayers as NN_encoder, and the numbers of nodes were 200, 400, and 2,000 from input, respectively.

In Kanade model, a predictor Pred_*l* is provided to predict the label *l* to be considered. The Pred_*l* is a linear predictor that predicts the label *l* from the set of elements $z_{i}^{\left(l\right)}$ of *z*_*i*_. Each $z_{i}^{\left(l\right)}$ has no common elements with each other. For the continuous label (time point of Continuous data and the ages of mice in the pancreatic atlas), the predictor functions as a regressor by computing a weighted sum of $z_{i}^{\left(l\right)}$, whereas for discrete labels (the other labels), it acts as a classifier by applying a softmax function to the weighted sum of $z_{i}^{\left(l\right)}$.

### Model training scheme

The VAE part and predictors of Kanade were simultaneously trained using the loss function below:

$$L_{\text{total}}=\alpha L_{\text{reconst}}+\beta L_{\text{KL}}+\sum_{l}\gamma_{l}L_{{\text{Pred}}_{l}}$$

Here, L_reconst_ is a reconstruction loss and is calculated as a negative log-likelihood. L_KL_ is a Kullback-Leibler divergence between prior and posterior probability of *z*_*i*_. $L_{{\text{Pred}}_{l}}$ is a prediction loss (cross entropy loss for discrete labels and mean squared error for continuous labels) of Pred_*l*. *α*, *β*, and *γ*_*l*_ are weights of each loss. The used weights in this study are listed in Table S1.

### Selection of dimensionality of each latent space and loss function weights

When the dimensionality of each latent space is excessive, the posterior distributions of some latent variables tend to approach a standard normal distribution with mean 0 and variance 1. This indicates that their posterior distributions are close to the prior distribution, suggesting that these latent variables contain little information. In this study, the dimensionality assigned to each label was adjusted to minimize the number of such uninformative latent variables.

In addition, inappropriate weights in the loss function can prevent specific loss terms from being learned, or cause some terms to be learned too rapidly, resulting in a large discrepancy between the loss values for the training and test data. In this study, we compared the loss values between the training and test data and selected settings that minimized overfitting. As examples, optimal and suboptimal settings for the Discrete Data are shown in Figure S24.

### Training for simulated datasets

The numbers of assigned elements of latent variables were one for the conditions, three for cell types, and five for batches. In our training, the minibatch size is 128, and the number of epochs is 25. Adam optimizer was used for all training, and the learning rate is 0.001. The dataset was split into training and test data at a 3:1 ratio, and parameters and seeds were selected for which the model had small overlearning.

### Preprocessing of mouse pancreatic islet scRNA-seq atlas data

The mouse pancreatic islet scRNA-seq atlas^17^ was downloaded from CELLxGENE database (id: 296237e2-393d-4e31-b590-b03f74ac5070) as a h5ad file. From the atlas, three datasets were excluded for the following reasons: “mSTZ” dataset had only one batch per condition, most of the cells in “Embryonic” dataset were annotated as “unknown”, and “Aged” dataset had a large gap of age from other datasets. As the units of ages of samples varied in the original annotations, all were standardized to months. As some annotations were made with wide ranges, they were assigned the midpoint values. Condition labels were added depending on a combination of pathogenic stresses (genotypes or chemicals) and treatments (Table S3).

Annotation of cell-type labels was performed by Seurat v4^8^. The original counts were loaded into a Seurat object, and the object was separated by the “donor_id” labels, which corresponded to batch identifiers. Separated objects were normalized by SCTransform^43^ function independently and merged again. The merged dataset was projected into the low dimensional space by RunPCA function, and 30 principal components (PCs) were used for RunUMAP function. PCs were corrected by Harmony.^9^ LISIs (see below) were calculated using 10, 30, and 50 Harmony-corrected dimensions, and we decided to use 10 dimensions for the clustering and UMAP projection because the majority of cells had LISI values larger than one. Clustering was performed by FindNeighbors and FindClusters function (“resolution” parameter was set to 0.1), and its results were used for Kanade training as cell-type labels. Marker genes of each cluster were detected by FindAllMarkers function, and each cluster was confirmed to correspond to a specific cell type. After annotations, genes that expressed only in less than 10 cells were excluded for the stable learning of models.

For the training, original counts were used. Before training, genes expressed in less than 10 cells were removed.

### Training for mouse pancreatic atlas

The numbers of assigned elements of latent variables were one for the age of mice, three for conditions, five for cell types, and 10 for batches (“donor_id” label). In our training, the minibatch size is 128, and the number of epochs is 50. Adam optimizer was used for all training, and the learning rate is 0.001. The dataset was split into training and test data at a 3:1 ratio, and parameters and seeds were selected for which the model had small overlearning.

### Preprocessing and training for human and mouse immune dataset

A batch obtained by Smart-seq2 was excluded from the dataset, and genes expressed in fewer than 10 cells were removed. The numbers of assigned elements of latent variables were one for species, one for tissue (samples from peripheral blood or bone marrow), five for cell types (“final_annotation” label), and 10 for batches. In our training, the minibatch size is 128, and the number of epochs is 50. Adam optimizer was used for all training, and the learning rate is 0.001. The dataset was split into training and test data at a 3:1 ratio, and parameters and seeds were selected for which the model had small overlearning.

### Preprocessing and training for SEA-AD dataset

The SEA-AD dataset was large, and data loading was time-consuming, making parameter search and downstream analyses impractical. Therefore, we randomly sampled 50,000 cells before analysis. We also removed genes expressed in fewer than 10 cells. The numbers of assigned elements of latent variables were one for disease, 10 for cell types (“Subclass” label), and 10 for batches (“donor_id” label). In our training, the minibatch size is 128, and the number of epochs is 100. Adam optimizer was used for all training, and the learning rate is 0.001. The dataset was split into training and test data at a 3:1 ratio, and parameters and seeds were selected for which the model had small overlearning.

### Generation of simulated datasets

To evaluate Kanade, two simulated datasets were generated. The first dataset had discrete conditions (Discrete data, Figure 2A). This dataset simulated an experiment comparing healthy wild-type and disease mutant. In the “Disease” condition, condition-dependent genes were up- or down-regulated compared with “WT.” This dataset contains two conditions, each with three batches, resulting in a total of six batches. The second dataset assumed that the expression of condition-dependent genes changes continuously over time, and cells were sampled at five time points (Continuous data, Figure S5A). Here, three batches were sampled at each time point, and 15 batches were obtained in total. Both datasets include three cell types (A, B, and C). Batch effects were randomly introduced in each batch (Figures 2A–2C and S5A–S5C).

To reproduce this setting, we assumed a total of 20,000 genes and categorized them as follows: marker genes for each cell type (mA, mB, and mC); genes upregulated in each cell type under disease conditions (or over time) (uA, uB, and uC); genes downregulated in each cell type under disease conditions (or over time) (dA, dB, and dC); and other non-variable genes (others). For simplicity, these categories have no overlap. The numbers of each gene type are listed in Table S2. Variable genes were randomly chosen from 15,000 genes with the largest basic mean-expressions, which are explained next.

To generate simulated datasets, the method of Splatter^44^ was modified. First, basic mean-expressions of 20,000 genes were sampled from a gamma distribution (shape = 0.6 and rate = 0.3). For outlier expression, genes were sampled from 20,000 genes using a binomial distribution with a mean 0.05, and the basic mean-expression of each sampled gene *g* was replaced by *m*_*g*,*out*_ defined below:

$$\Psi_{g}∼\ln\;\text{Normal}\left(\mu_{out},\sigma_{out}\right)$$

$$m_{g,out}=\Psi *m_{median}$$

Here, *μ*_*out*_ = 4 and *σ*_*out*_ = 0.5 were used, and *m*_*median*_ was the median value of basic mean-expression of all genes before the replacement.

To introduce cell-type-dependent variation, the magnitude of variation for a variable gene *g* was sampled by

$$v_{\text{type},g}∼\ln\;\text{Normal}\left(\mu_{\text{type}},\sigma_{\text{type}}\right)$$

Here, *μ*_type_ = 2 and *σ*_type_ = 0.5. If *v*_type,*g*_ < 1.1, the value was replaced by 1.1 to ensure a minimum variation threshold.

• For marker genes and downregulated genes, the mean expression of *g* was *v*_type,*g*_ folded in the corresponding cell type and 1/*v*_type,*g*_ folded in the other cell types.
• For upregulated genes, the mean expression was 1/*v*_type,*g*_-fold in the corresponding cell type.

To account for condition-dependent variation, magnitudes of variation for a condition-dependent gene *g* was sampled by

$$v_{\;\text{condition},g}∼\ln\;\text{Normal}\left(\mu_{\text{condition}},\sigma_{\text{condition}}\right)$$

Here, *μ*_condition_ = 2 and *σ*_condition_ = 0.5. If *v*_condition,*g*_ < 1.1, the value was replaced by 1.1 to ensure a minimum variation threshold.

• For Discrete data:
– If *g* was the upregulated gene, its mean expression was *v*_condition,*g*_ folded in the corresponding cell type in the disease condition.
– If *g* was the downregulated gene, its mean expression was 1/*v*_condition,*g*_ folded.
• For Continuous data:
– If *g* was the upregulated gene, its mean expression of upregulated at time point *t* was $v_{\text{condition},g}^{t}$ folded
– If *g* was the downregulated gene, its mean expression of upregulated at time point *t* was $v_{\text{condition},g}^{t}$ folded, and if the downregulated gene $v_{\text{condition},g}^{-t}$ folded.

For a representation of batch effects, random perturbations were applied to the mean expression levels of genes for each batch. Specifically, for batch *b* and gene *g*, *c*_*g*,*b*_ was randomly assigned such that it took the value 0 with a probability of 0.8, 1 with a probability of 0.1, and −1 with a probability of 0.1. Additionally, the magnitude of the batch effect of gene *g* was sampled from a log-normal distribution:

$$v_{\text{batch},g,b}∼\ln\;\text{Normal}\left(\mu_{\text{batch}},\sigma_{\text{batch}}\right)$$

Here, *μ*_batch_ = ln 2 and *σ*_batch_ = 0.12. If *v*_batch,*g*,*b*_ was less than 1.1, it was replaced with 1.1 to ensure a minimum variation threshold. Using these values, the mean expression level of gene *g* was $v_{\text{batch},g,b}^{c_{g,i}}$ folded. For the simulated dataset without batch effects, this step was skipped.

To capture single-cell variability, the following procedure was implemented. Let $m_{g}^{d,\Gamma }$ denote the mean expression level of gene *g* under condition d in cell type Γ(∈ {*A*, *B*, *C*}), as defined above. First, the library size ${\hat{s}}_{i}$ for each cell *i* was sampled using the following equation:

$${\hat{s}}_{i}∼\ln\;\text{Normal}\left(\mu_{\text{libsize}},\sigma_{\text{libsize}}\right)$$

Here, *μ*_libsize_ = ln 5000 and *σ*_libsize_ = 0.2. Using these values, the expression level $m_{g,i}^{d,\Gamma }$, adjusted for the library size of each cell, was computed as follows:

$$m_{g,i}^{d,\Gamma }=\frac{{\hat{s}}_{i}m_{g}^{d,\Gamma }}{\sum_{g}m_{g}^{d,\Gamma }}$$

True expression value of gene *g* in each cell ${\tilde{m}}_{g,i}^{d,\Gamma }$ was obtained by the following:

$$\text{dof}∼\chi^{2}\left({\text{df}}_{0}\right)$$

$${\text{BCV}}_{g,i}=\left(\phi +\frac{1}{\sqrt{m_{g,i}^{d,\Gamma }}}\right){\left(\frac{{\text{df}}_{0}}{\text{dof}}\right)}^{1/2}$$

$${\tilde{m}}_{g,i}^{d,\Gamma }∼\text{Gamma}\left(\frac{1}{{\text{BCV}}_{g,i}^{2}},m_{i,k}^{d,\Gamma }{\text{BCV}}_{g,i}^{2}\right)$$

Here, df_0_ = 60 and *ϕ* = 0.1. This process was performed for each batch and cell type.

To simulate RNA-seq sampling, Poisson distribution was used:

$$y_{g,i}∼\text{Poisson}\left({\tilde{m}}_{g,i}^{d,\Gamma }\right)$$

Here, *y*_*g*,*i*_ was the true count of a gene *g* in a cell *i*. Usually, small library sizes of scRNA-seq result in dropout, where some gene count values are artificially recorded as zero despite the gene being expressed. Thus, the observed value *x*_*g*,*i*_ was obtained as follows:

$$\pi_{g,i}=\frac{1}{1+\exp\left\{-a\left(\ln\;{\tilde{m}}_{g,i}^{d,\Gamma }-x_{0}\right)\right\}}$$

$$h_{g,i}∼\text{Bernoulli}\left(1-\pi_{g,i}\right)$$

$$x_{g,i}=\left\{ \begin{array}{cc} y_{g,i} & \text{if}\;h_{g,i}=1\\ 0 & \text{if}\;h_{g,i}=0 \end{array}\right.$$

The parameters were set to *a* = −1 and *x*_0_ = 0.

### Count reconstruction and downstream dimensionality reduction

For simulated data, uncorrected counts were input to the trained encoder and projected to latent variables. After latent variables for the batches were replaced with zeros, ZINB parameters were obtained from latent variables by the trained decoder. Among these parameters, the scale factors were averaged, and counts were reconstructed based on the obtained ZINB parameters. For the pancreatic atlas, in addition to the replacement of latent variables for the batches, latent variables for the ages were averaged before decoding.

Downstream analysis was performed by Seurat v.4. The reconstructed counts were log-transformed after the addition of 1. Using FindVariableFeatures function, 3,000 highly variable genes (HVGs) were detected. After reconstructed counts of HVGs were standardized with ScaleData function, PCA was performed with RunPCA functions. UMAP was performed with RunUMAP function and the top 30 PCs. For the analysis of beta cells in the pancreatic atlas, cells with the label “beta” of the metadata “cell_type_reannotatedIntegrated” by Hrovatin et al. after the analysis above were used. PCA was re-performed on the extracted beta cells.

### Differentially expressed gene analysis

DEG analysis was performed by FindMarkers function in Seurat v.4. The parameter test.use was set to “MAST.”^45^ For simulated datasets, thresholding parameters “min.pct” and “logfc.threshold” were set to 0 so that all genes were considered. For comparison between control and NOD in the mouse pancreatic atlas, to avoid an excessive number of cells, we randomly selected 3,000 beta cells from each of the control and NOD conditions before analysis. Thresholding parameters “min.pct” and “logfc.threshold” were set to 0 so that all genes were considered. Genes were ranked in ascending order of *p* value, and for the top 200 genes identified using uncorrected counts, we examined the change in rank after correction. For the comparison between db/db and db/db_VSG conditions in mouse pancreatic atlas, “logfc.threshold” was set to 0 and “min.pct” default value (0.01).

### Gene ontology term enrichment analysis

In the PCA of beta cells in the pancreatic atlas, the 100 genes with the smallest “feature.loading” values for PC 2 and PC 3 were listed respectively and used for the gene ontology (GO) term enrichment analysis. For GO term enrichment analysis, g:Profiler (https://biit.cs.ut.ee/gprofiler)^46^ was used with g:SCS algorithm, and significantly enriched terms in cellular components were identified at a false discovery rate (FDR) threshold of 0.05 and term_size lower than 5,000.

### Calculation of local inverse Simpson’s indices

Preprocessing was performed by Seurat v.4. and Harmony. Simulated datasets without batch effects and simulated uncorrected datasets with batch effects were normalized and scaled by NormalizeData function and ScaleData function, with 3,000 HVGs detected by FindVariableFeatures function. Harmony was applied to the top 30 PCs of uncorrected datasets. The preprocessing of reconstructed data was explained above. For PCA space and Harmony space, the top 30 dimensions in PCs or Harmony-corrected variables were used for the calculation of LISIs. For latent space of VAE, all dimensions were used for the calculation of LISIs. LISIs were calculated by the compute_lisi function in R package lisi.^9^

### Application of conventional VAE

scVI^11^ was performed according to the official tutorial. For Discrete data, the parameter search for n_hidden, n_latent, and n_layers was performed, and n_hidden = 2048, n_latent = 10, and n_layers = 2 were used for Figure 2. For all other scVI analyses, the default parameters were used. The Batch ID was set to “categorical_covariate_keys” for all scVI analyses.

In scArches analyses, TRVAE model was trained, according to the official tutorial. For Discrete data, the subset with “WT” labels was used as a reference, and the model was re-trained by using the subset of cells labeled “Disease” as a query. For mouse pancreatic data, the subset with “control” label was used as reference and others were used as queries. For human and mouse immune dataset, subsets from human and peripheral blood were used as reference and others were used as query.

SCDC^15^ analyses were performed according to the code on the GitHub page by its authors. The n_type parameter was set to the product of the number of cell types and the number of condition combinations considered in Kanade. All other parameters were kept at their default values.

For the scVI, scArches, and SCDC latent space, dimensionality reduction was performed on each latent space by using tl.umap functions in Scanpy package.

### Calculation of RNA velocity

For calculation of RNA velocity, scvelo package^41^ was used. The mouse gastrulation dataset^40^ was downloaded by scvelo.datasets.gastrulation function. Using original counts, the preprocessing was performed according to official tutorial of the scvelo package. In this process, 2,000 highly variable genes (HVGs) were detected. Based on the HVGs expression, RNA velocity was calculated using the tl.velocity function. For Kanade correction, the same HVG set was used. Spliced and unspliced counts were merged before application of Kanade. In the Kanade training, the minibatch size is 128, and the number of epochs is 50. Adam optimizer was used with the learning rate at 0.001. The dataset was split into training and test data at a 3:1 ratio. After reconstructed counts were separated into spliced and unspliced counts, RNA velocity was calculated in the same way as for the original counts.

## Resource availability

### Lead contact

Requests for further information and resources should be directed to and will be fulfilled by the lead contact, Shunta Sakaguchi (sakaguchi.shunta.g5@f.mail.nagoya-u.ac.jp).

### Materials availability

This study did not generate new unique reagents.

### Data and code availability

All code and simulated datasets for the analysis are available at our Zenodo repository (https://doi.org/10.5281/zenodo.20151245).^47^ The mouse pancreatic islet scRNA-seq atlas by Hrovatin et al. and SEA-AD are available from CELLxGENE database (id: 296237e2-393d-4e31-b590-b03f74ac5070 and 92b37feb-aa2c-40d7-bd90-0a9b5ddb3b27). Mouse and human immune data are available from Figshare (https://doi.org/10.6084/m9.figshare.12420968). The mouse gastrulation dataset is available at Figshare (https://doi.org/10.6084/m9.figshare.14627496.v1). The Python package version of Kanade is available from the GitHub page (https://github.com/ShuntaSakaguchi/kanade).

## Acknowledgments

This research was supported by the Moonshot R&D–MILLENNIA Program (JPMJMS2024-9 to H.N.) and 10.13039/501100003382CREST (JPMJCR25Q2 to H.N.) from the 10.13039/501100002241Japan Science and Technology Agency, 10.13039/100009619Japan Agency for Medical Research and Development Multidisciplinary Frontier Brain and Neuroscience Discoveries (Brain/MINDS 2.0) (JP25wm0625322 to H.N.), 10.13039/501100001691JSPS KAKENHI (26K21377 to S.S.), and Takeda Science Foundation Medical Research Grant (2025046603 to M.T.). We would like to thank Shusei Fujioka for valuable advice on marker genes of immune cells, Ryosuke Kojima for advice on the model development, and the members of Honda Lab and other laboratories of the mathematical biology group at Hiroshima University for their insightful discussion. Additionally, we acknowledge the assistance of ChatGPT in coding and manuscript writing.

## Author contributions

S.S., M.T., K.N., and H.N. conceived the model. S.S. and M.T. developed the software. S.S. performed validations and applied the model to real data. S.S., M.T., K.N., and H.N. wrote the manuscript. H.N. supervised the work.

## Declaration of interests

The authors are inventors on a pending Japanese patent application (Japanese Patent Application No. 2024-162597) filed by Hiroshima University that covers aspects of the technology described in this manuscript.

## Declaration of generative AI and AI-assisted technologies in the writing process

During the preparation of this work, the authors used ChatGPT in order to receive assistance with coding and English-language manuscript preparation. After using this service, the authors reviewed and edited the content as needed and take full responsibility for the content of the publication.

## Supplemental information

Document S1. Figures S1–S24

Table S1. List of weights of loss function for each training

Table S2. The number of each type of genes in simulated datasets

Table S3. Batch information of mouse pancreatic dataset

Table S4. Accuracy of the prediction of each label from each latent variable

Document S2. Article plus supplemental information
