# Supplementary material for: Disentangling batch effects and biological signals across conditions in single-cell transcriptome
Source: Patterns (N Y). 2026 Aug 19;7(10):101638. doi: 10.1016/j.patter.2026.101638 (PMC13674314; doi:10.1016/j.patter.2026.101638)
Supplement: Supplementary file 1 [file mmc1.pdf]

Patterns, Volume *7*

Supplemental information

Disentangling batch effects and biological signals across conditions in single-cell transcriptome

Shunta Sakaguchi (坂口峻太), Masato Tsutsumi (堤真人), Kentaro Nishi, and Honda Naoki

Supplementary Figures

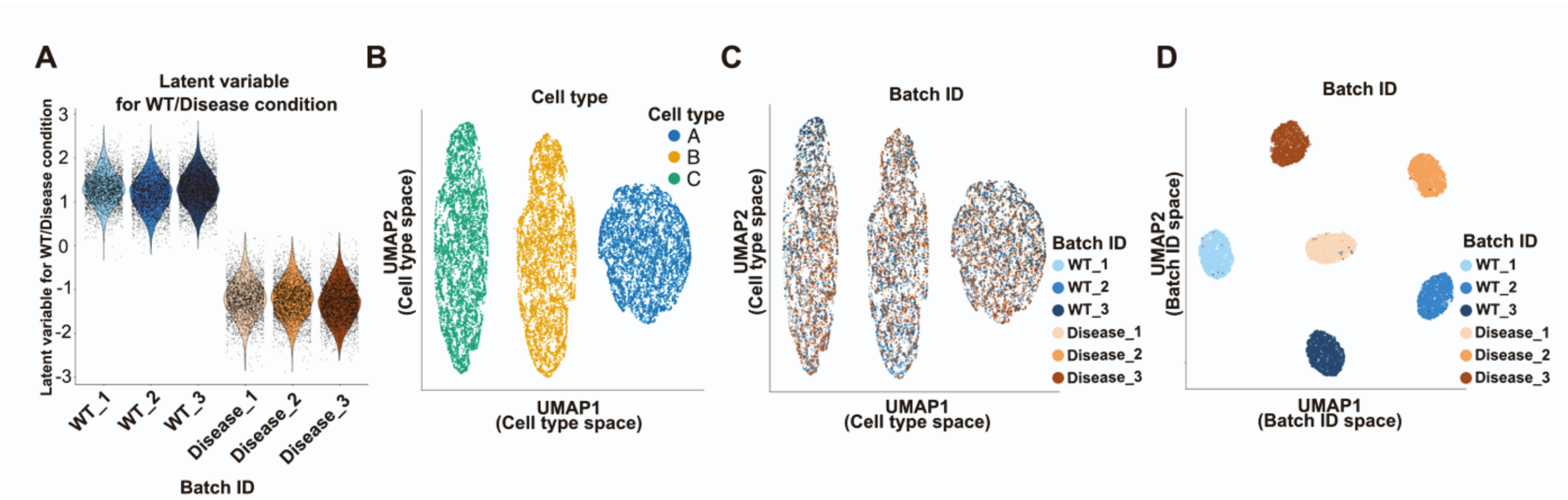

Figure S1: **Descrete Data in specialized latent spaces**
(A) The violin plots of the latent variable for WT/Disease condition across independent batches. Each dot represents an individual cell.
(B and C) UMAP plots of discrete data using latent variables for cell types. Colors of dots show cell types (B) and batch IDs (C).
(D) UMAP plot of discrete data using latent variables for batches. Colors of dots show batch IDs.

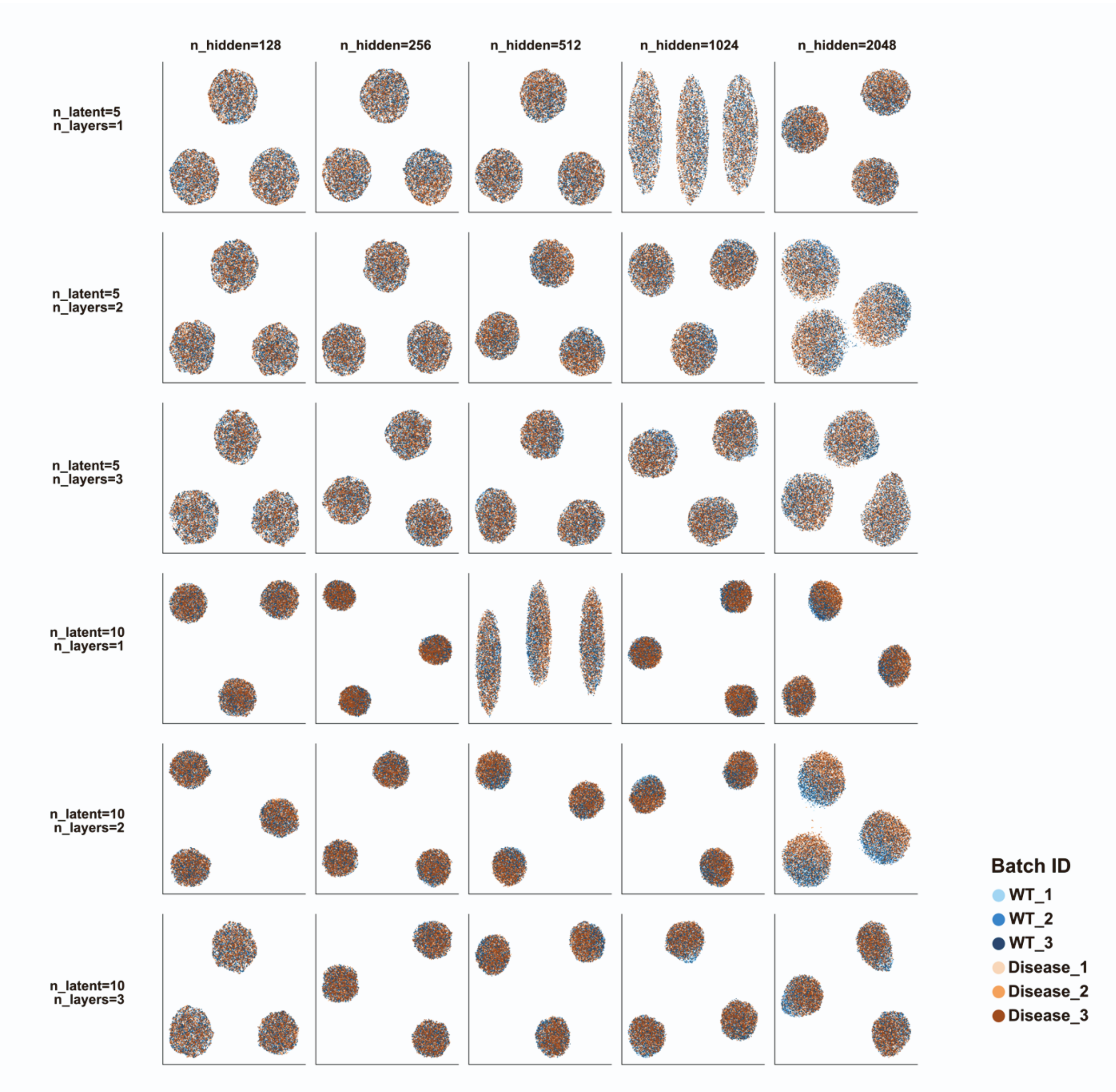

Figure S2: **Latent variables of scVI with various hyperparameters for Discrete Data**
The UMAP plots of scVI with various hyperparameters. Colors of dots show Batch IDs. Parameters, n_hidden, n_latent, n_layers mean the number of nodes in hidden layers, that of latent variables and that of hidden layers.

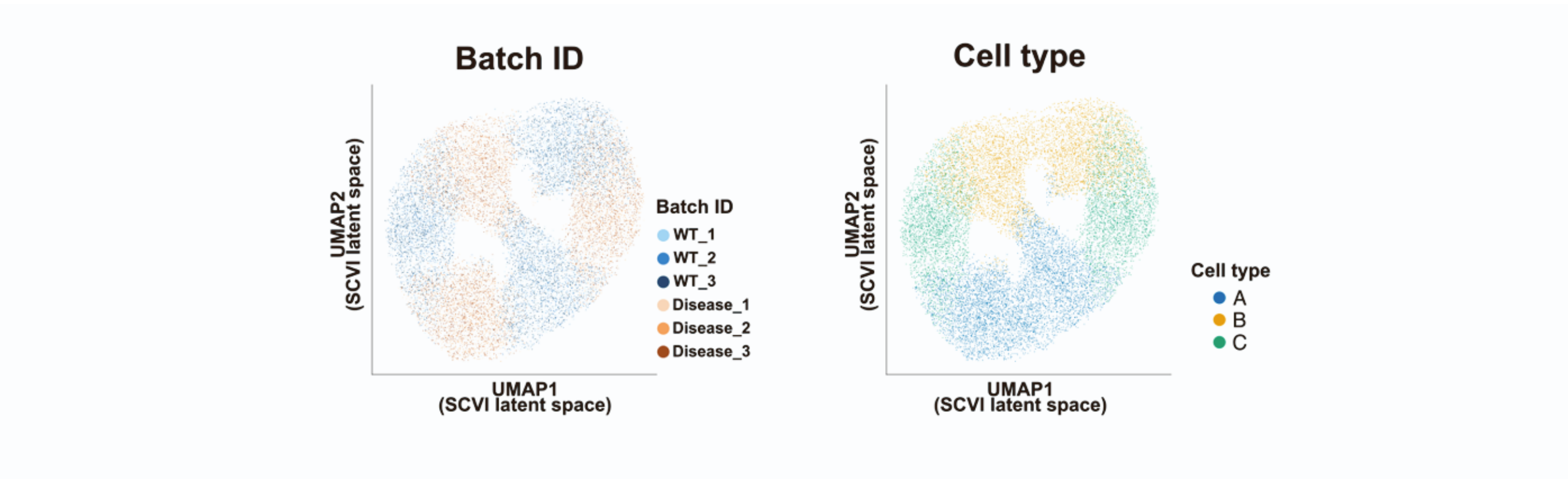

Figure S3: **Merged latent variables of scVI for Discrete Data**
UMAP plot of the merged latent space obtained by applying scVI separately to each condition. Colors of dots show Batch IDs.

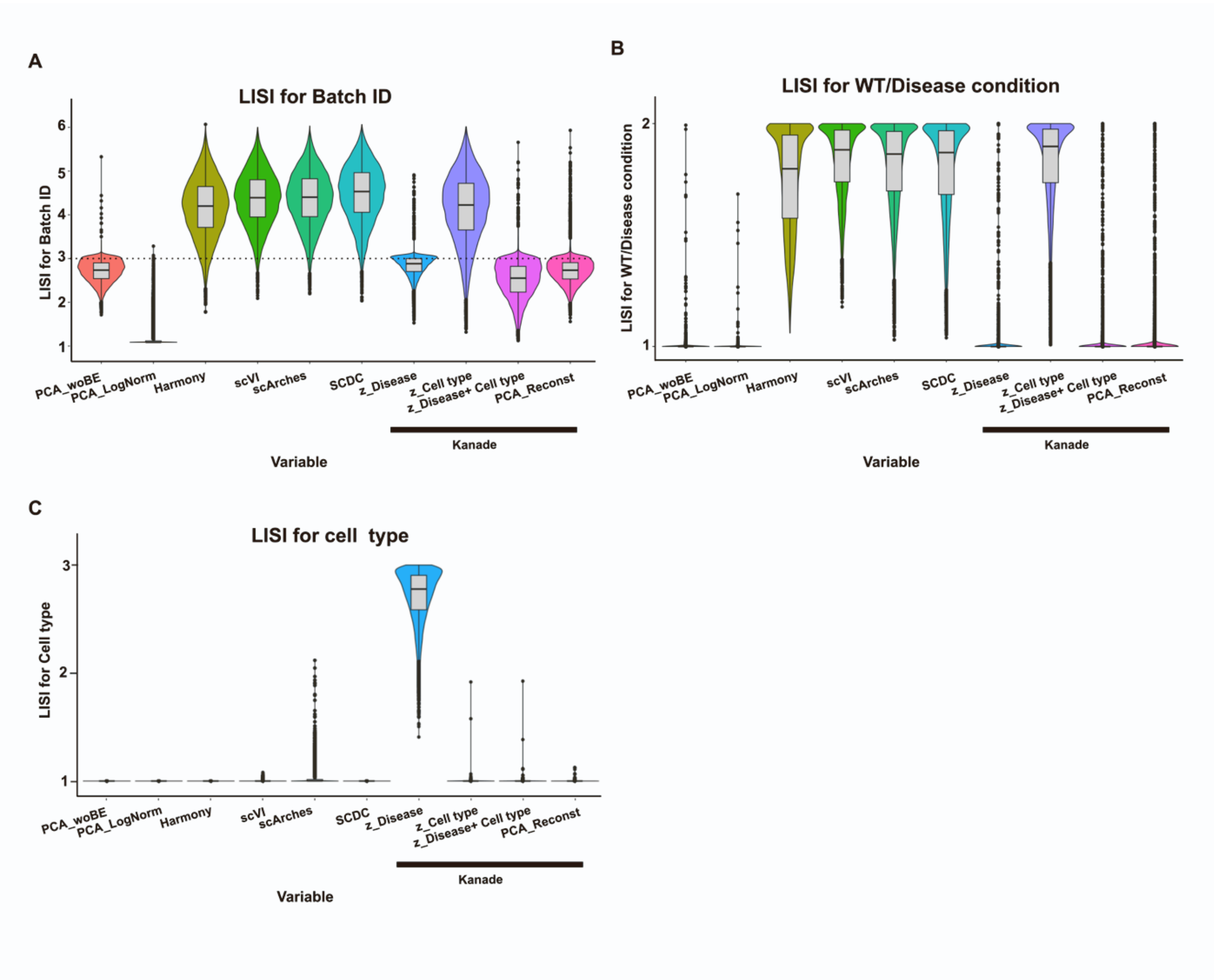

Figure S4: **Quantitative validation by LISI values for Discrete Data**
The violin plots and box plots of LISI for Batch ID (A), WT/Disease conditions (B) and cell types (C). Dots represent outliers in box plots.

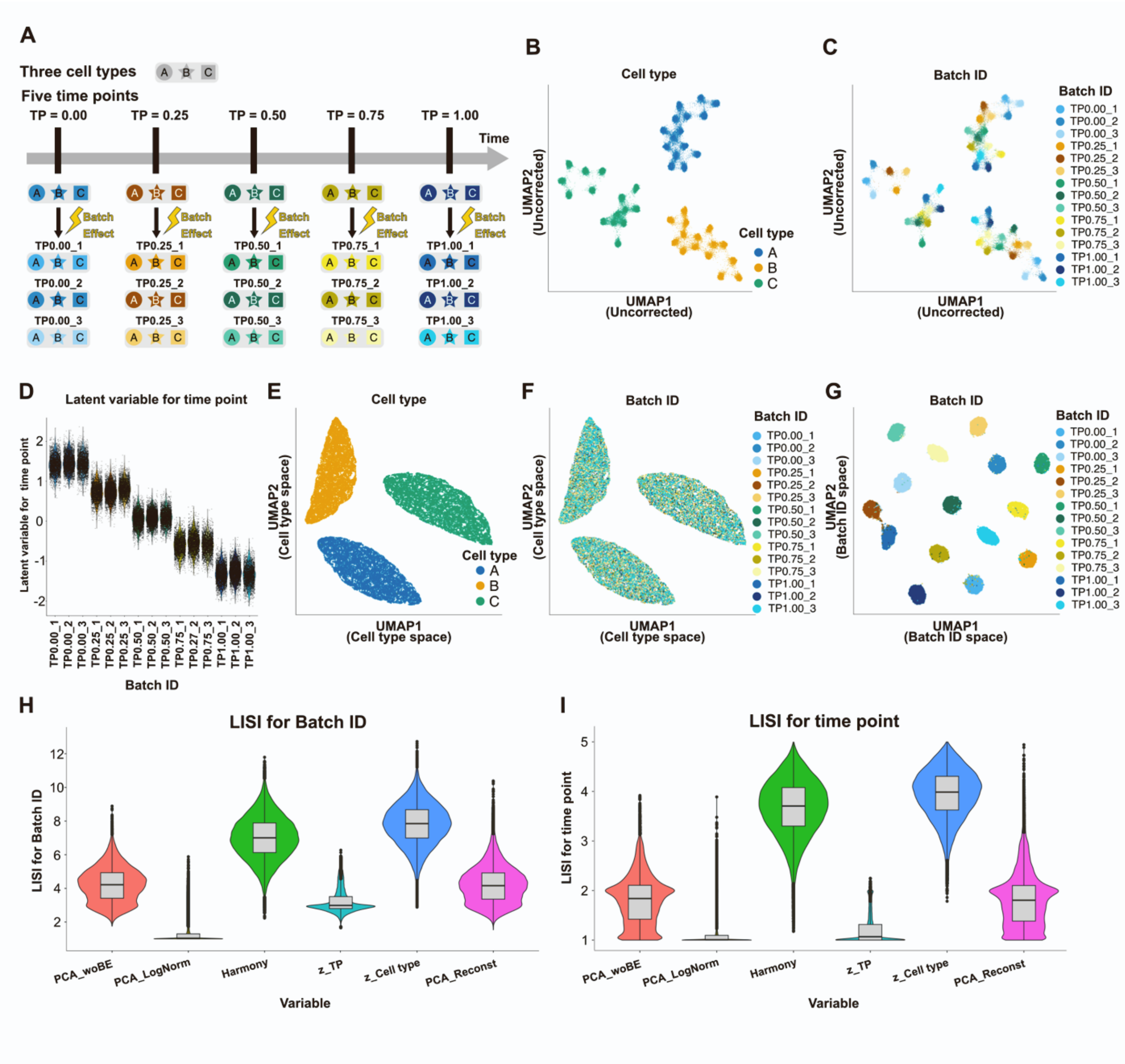

Figure S5: **Simulated continuous data in the latent variable spaces of Kanade**
(A) The scheme of Continuous data.
(B and C) UMAP plots of continuous data based on log-normalization. The colors of the dots show cell types (B) and batch IDs (C).
(D) The violin plots of the latent variable for time points across independent batches. Each dot represents an individual cell.
(E and F) UMAP plots of continuous data using latent variables for cell types. The colors of the dots show cell types (E) and batch IDs (F).

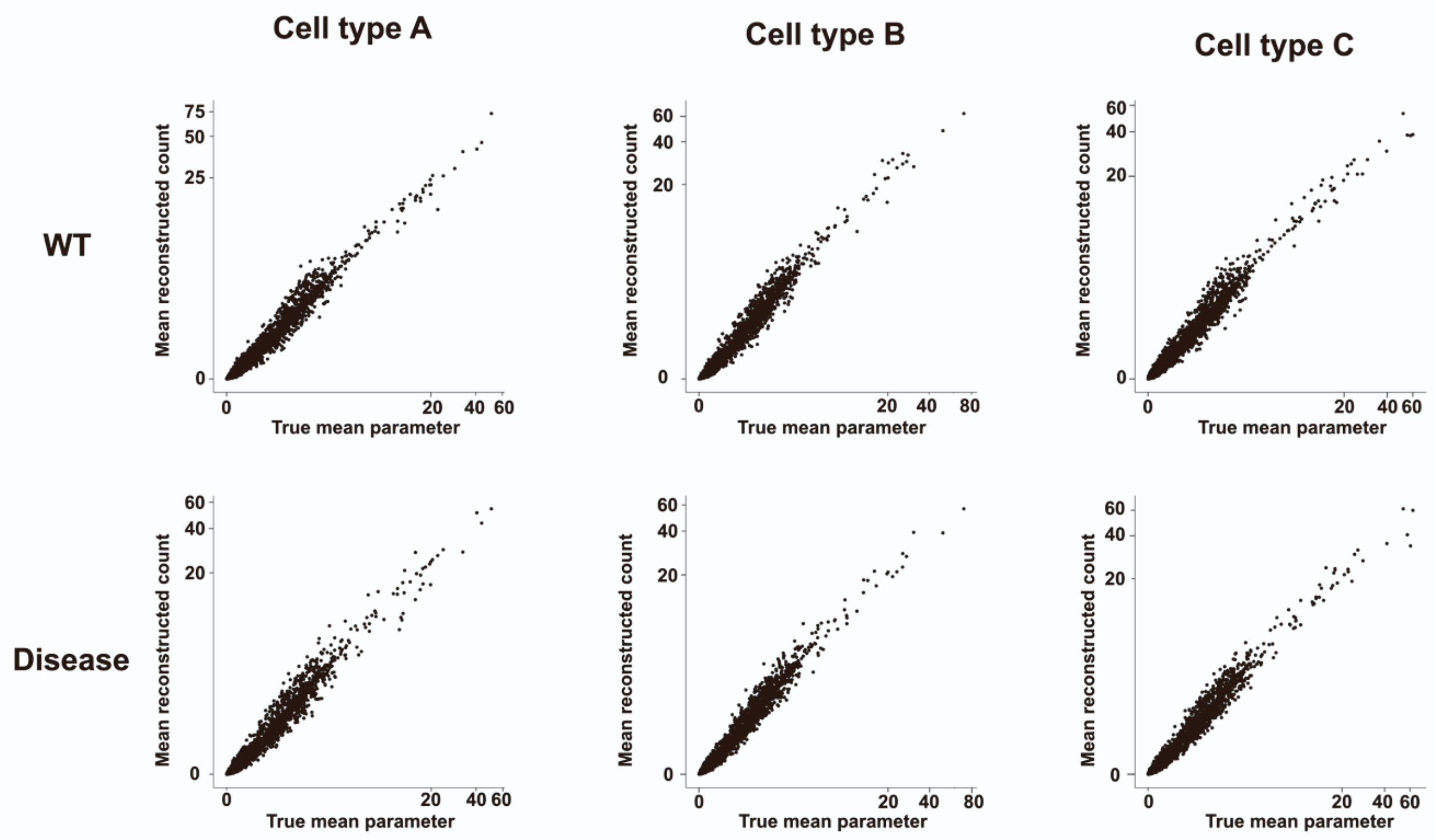

Figure S6: **Comparison of reconstructed counts and simulation parameters**
Scatter plots of mean gene expressions in each cell type and condition (Splitted version of Figure 3 by condition and cell type). The X -axis represents the mean parameters of the simulation and the Y-axis mean of reconstructed counts. Both axes are log-scaled. Each dot represents each gene.

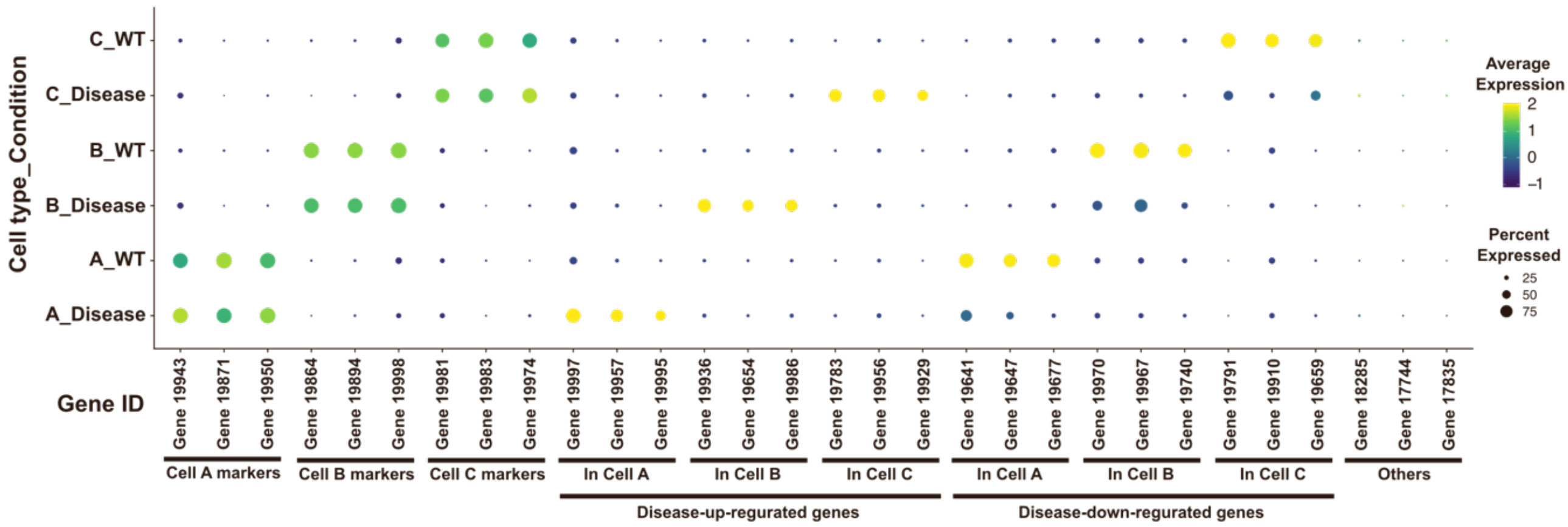

Figure S7: **Expression of marker genes and DEGs in reconstructed counts in Discrete Data**
The dot plot of reconstructed gene expressions in each cell type and condition.

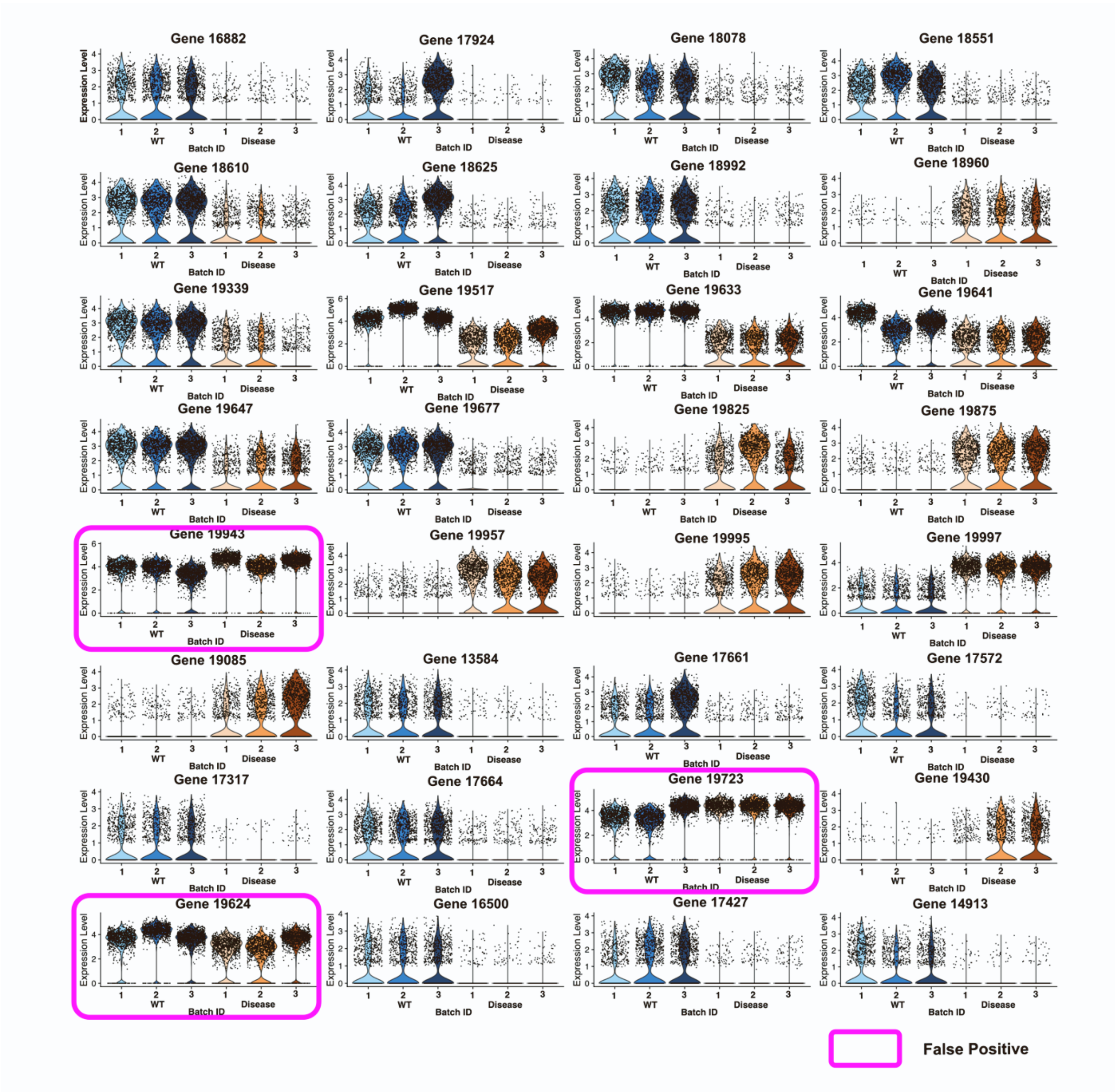

Figure S8: **Top 32 DEGs identified from uncorrected counts, plotted using uncorrected counts**

The violin plots of uncorrected counts of 32 DEGs with smallest p-values in the DEG analysis using uncorrected counts for Cell type A between conditions.

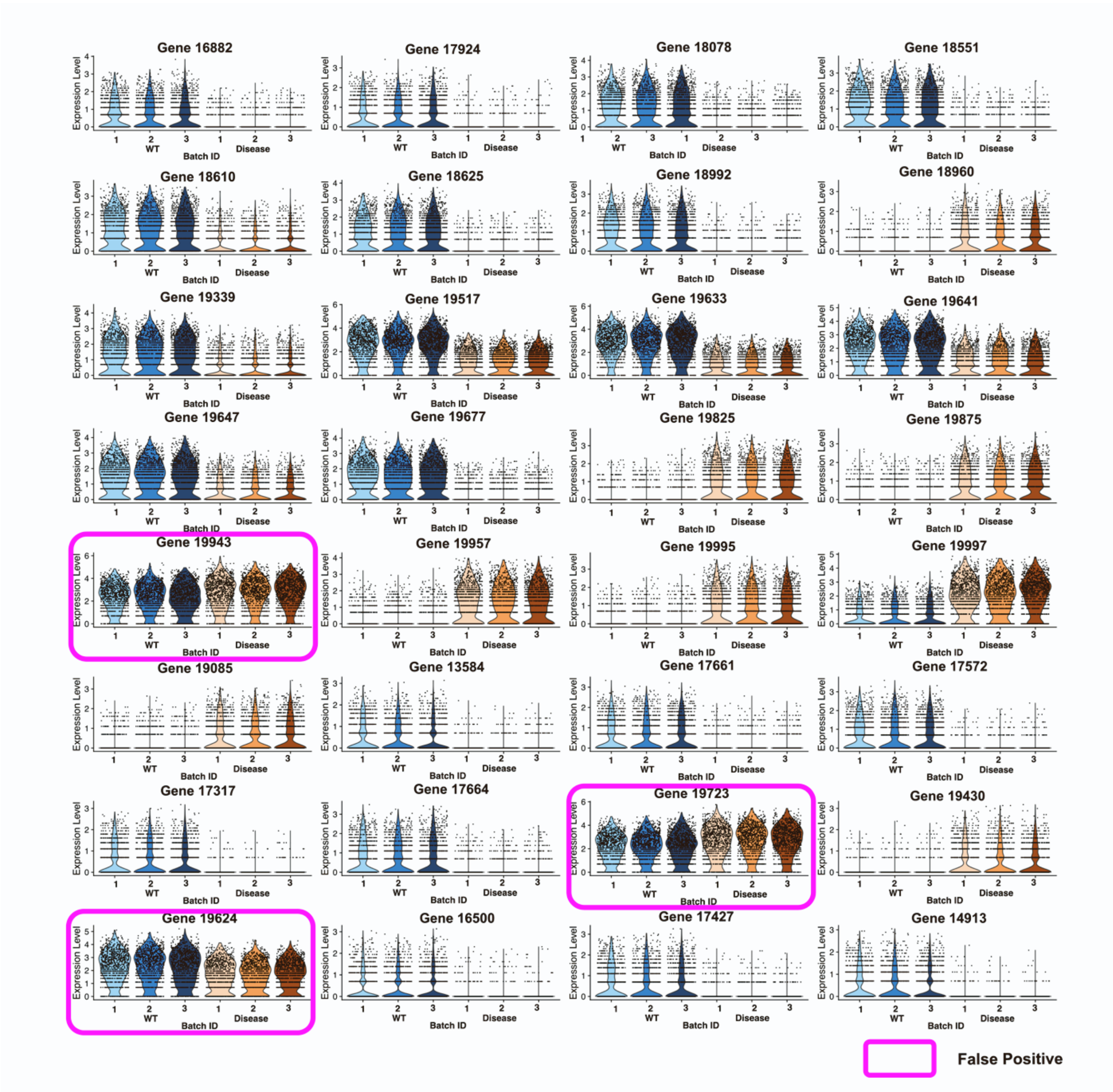

Figure S9: **Top 32 DEGs identified from uncorrected counts, plotted using Kanade-corrected counts**
The violin plots of Kanade-corrected counts of 32 DEGs with smallest p-values in the DEG analysis using uncorrected counts for Cell type A between conditions.

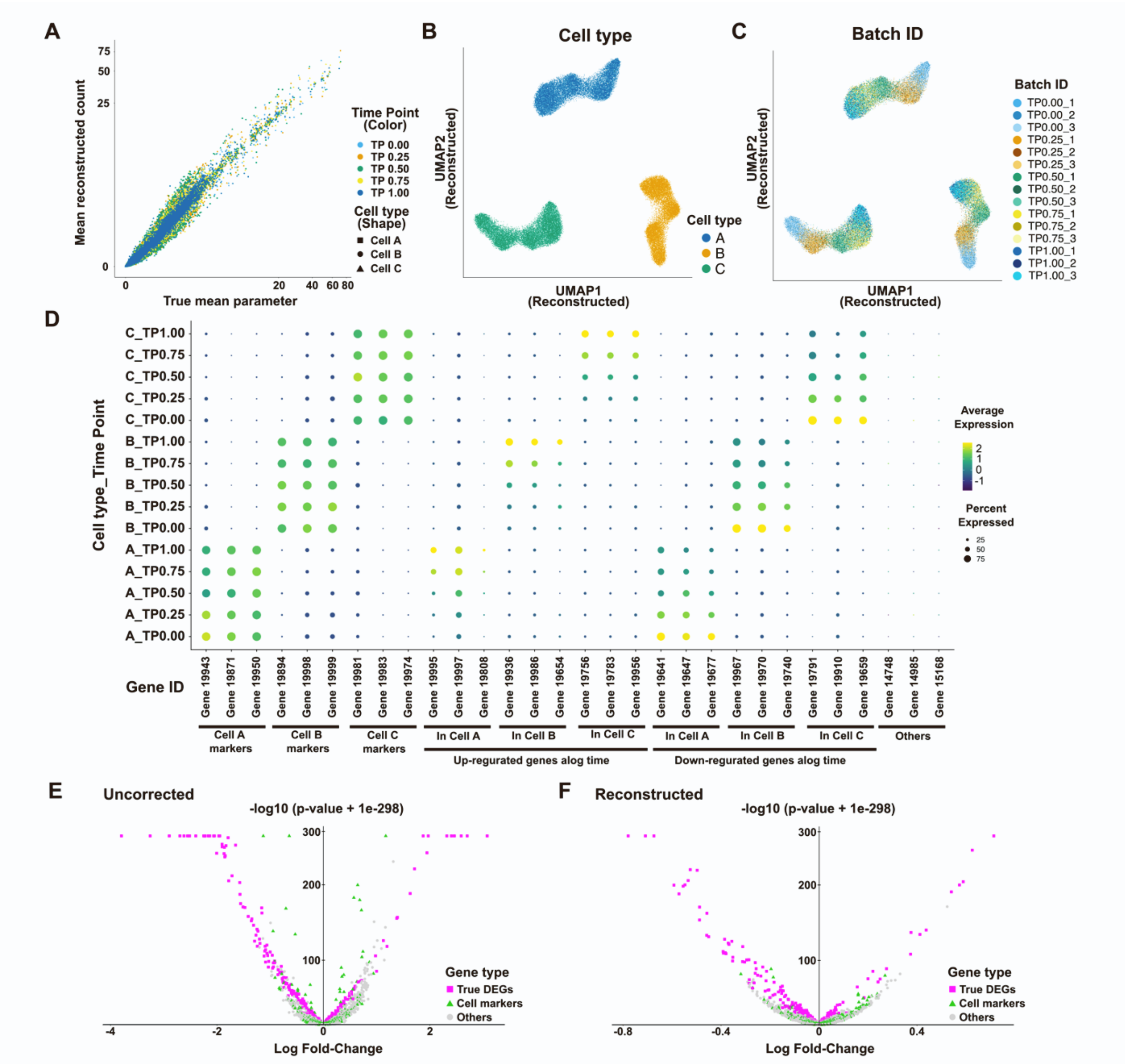

Figure S10: **Reconstructed counts of simulated continuous data**
(A) Scatter plots of mean gene expressions in each cell type and time point. The X-axis represents the mean parameters of the simulation and the Y-axis mean of reconstructed counts. Both axes are log-scaled. Each dot represents each gene, and the color and shape of the dots represent conditions and cell types respectively.
(B and C) UMAP plots of continuous data based on reconstructed counts. The colors of the dots show cell types (B) and batch IDs (C).
(D) The dot plot of reconstructed gene expressions in each cell type and condition.
(E and F) Volcano plots of differentially expressed gene analysis results in cell type A between time points 0.0 and 1.0 based on uncorrected log-normalization (E) and reconstructed counts by Kanade (F). Each dot means a gene and X-axis represents log2-fold changes and Y-axis negative log10 p-values. Small values (1e-298) are added into p-values to avoid the underflow.

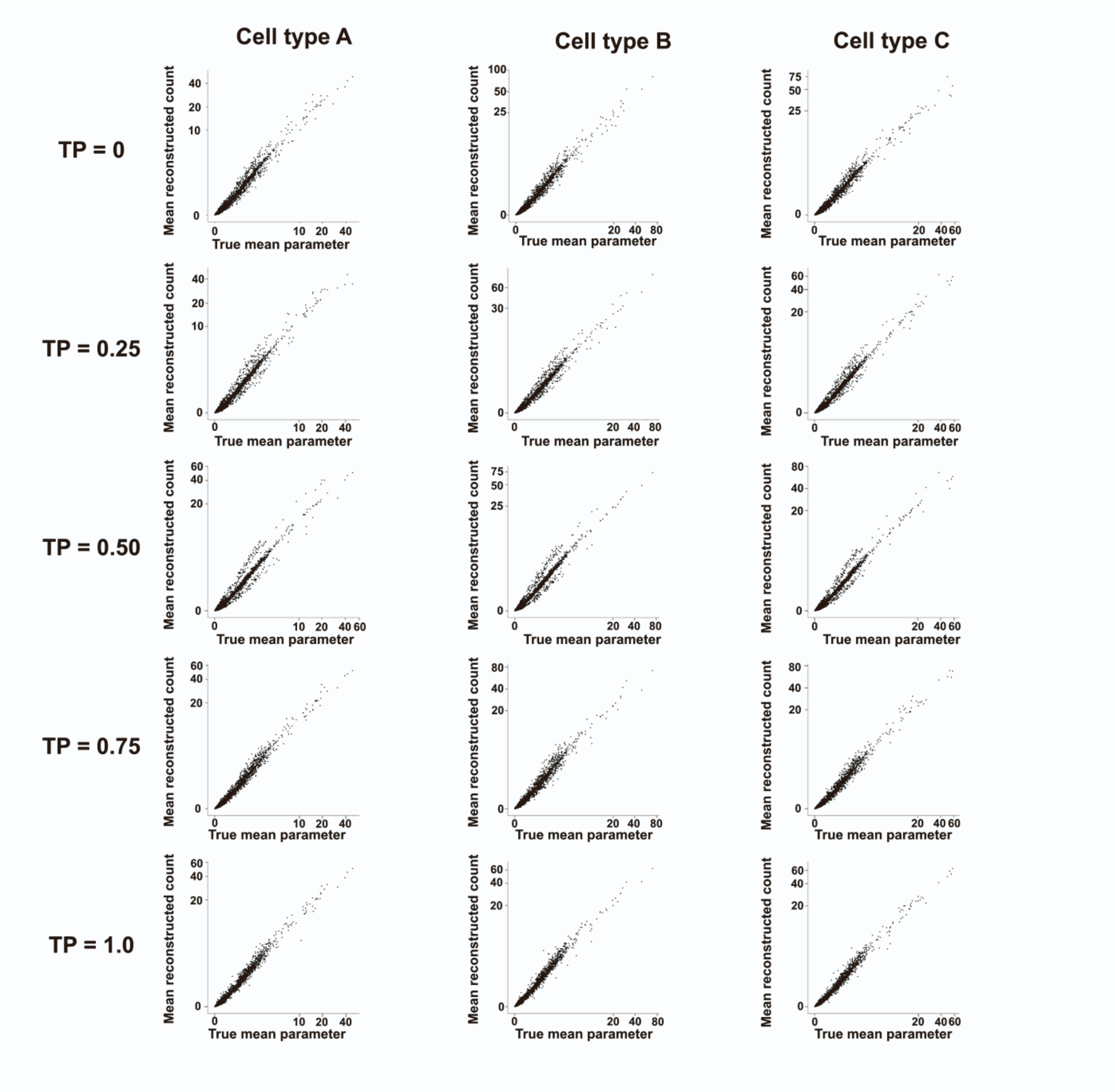

Figure S11: **Comparison of reconstructed counts and simulation parameters**
Scatter plots of mean gene expressions in each cell type and condition (Splitted version of Figure S10 by conditon and cell type). The X -axis represents the mean parameters of the simulation and the Y-axis mean of reconstructed counts. Both axes are log-scaled. Each dot represents each gene.

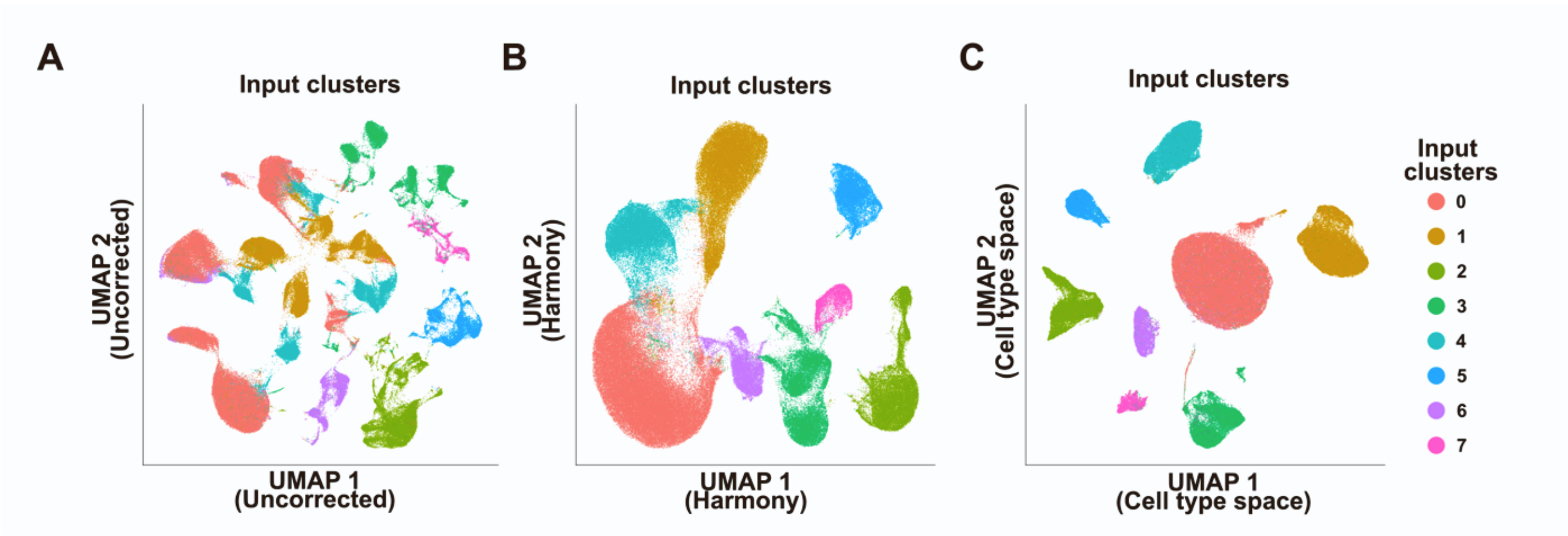

Figure S12: **Input clusters of mouse pancreatic atlas for the training**
Input clusters of mouse pancreatic atlas for the training of Kanade were shown with the color of dots on the UMAP plots based on log-normalization (A), Harmony-corrected (B), and latent variables for cell types of Kanade (C).

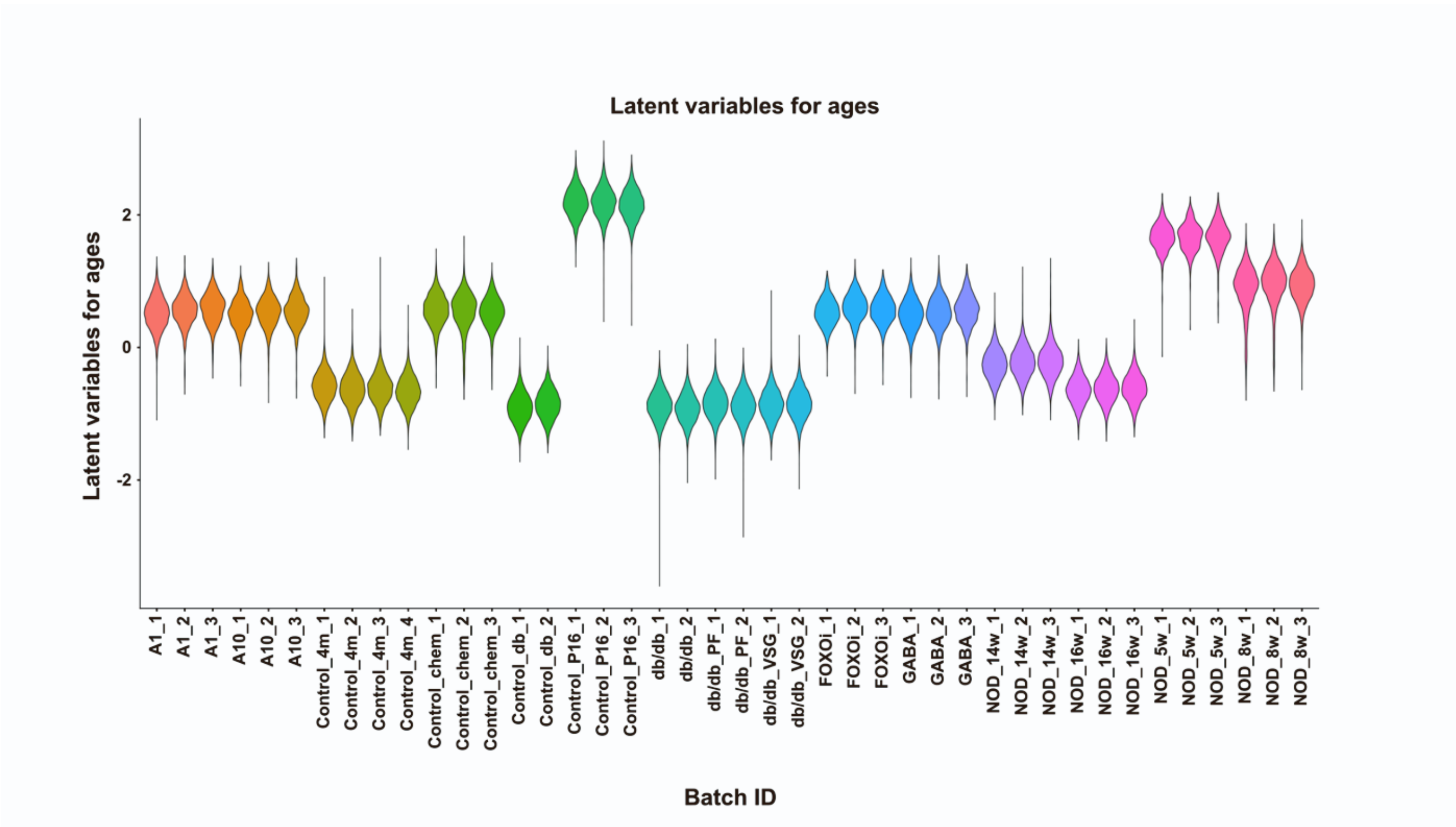

Figure S13: **Latent variable for the ages of mice across the batches**.
Violin plots of the latent variable for the ages of mice across the batches.

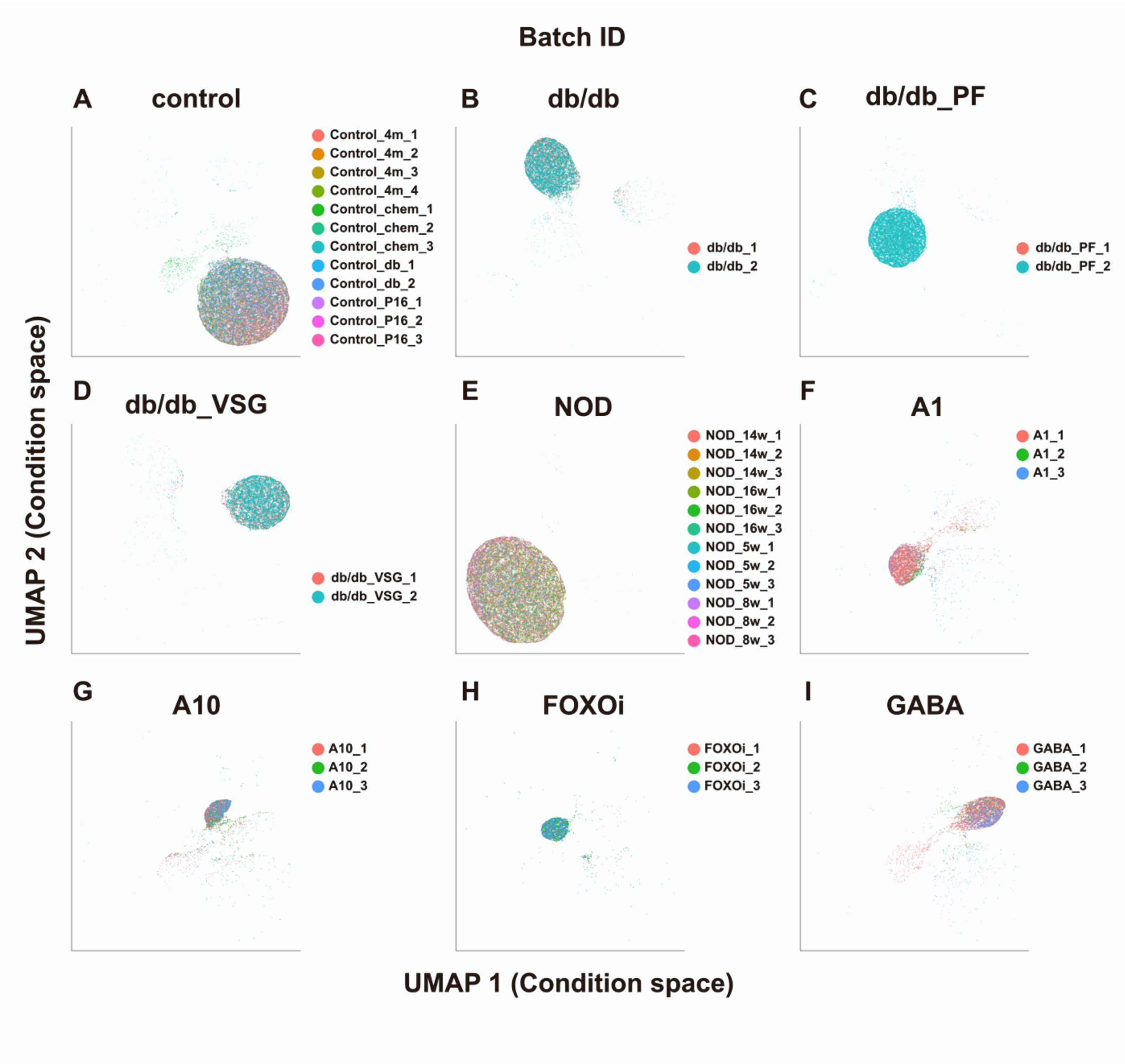

Figure S14: **Batches on latent variables for conditions**
UMAP plots of the mouse pancreatic atlas based on latent variables for conditions. The colors of the dots show batches, and plots were separated by conditions. (A) control, (B) db/db, (C) db/db_PF, (D) db/db_VSG, (E) NOD, (F) A1, (G) A10, (H) FOXOi and (I) GABA
A1 and A10: artemether (1 or 10 μM); FOXOi: FoxO inhibitor

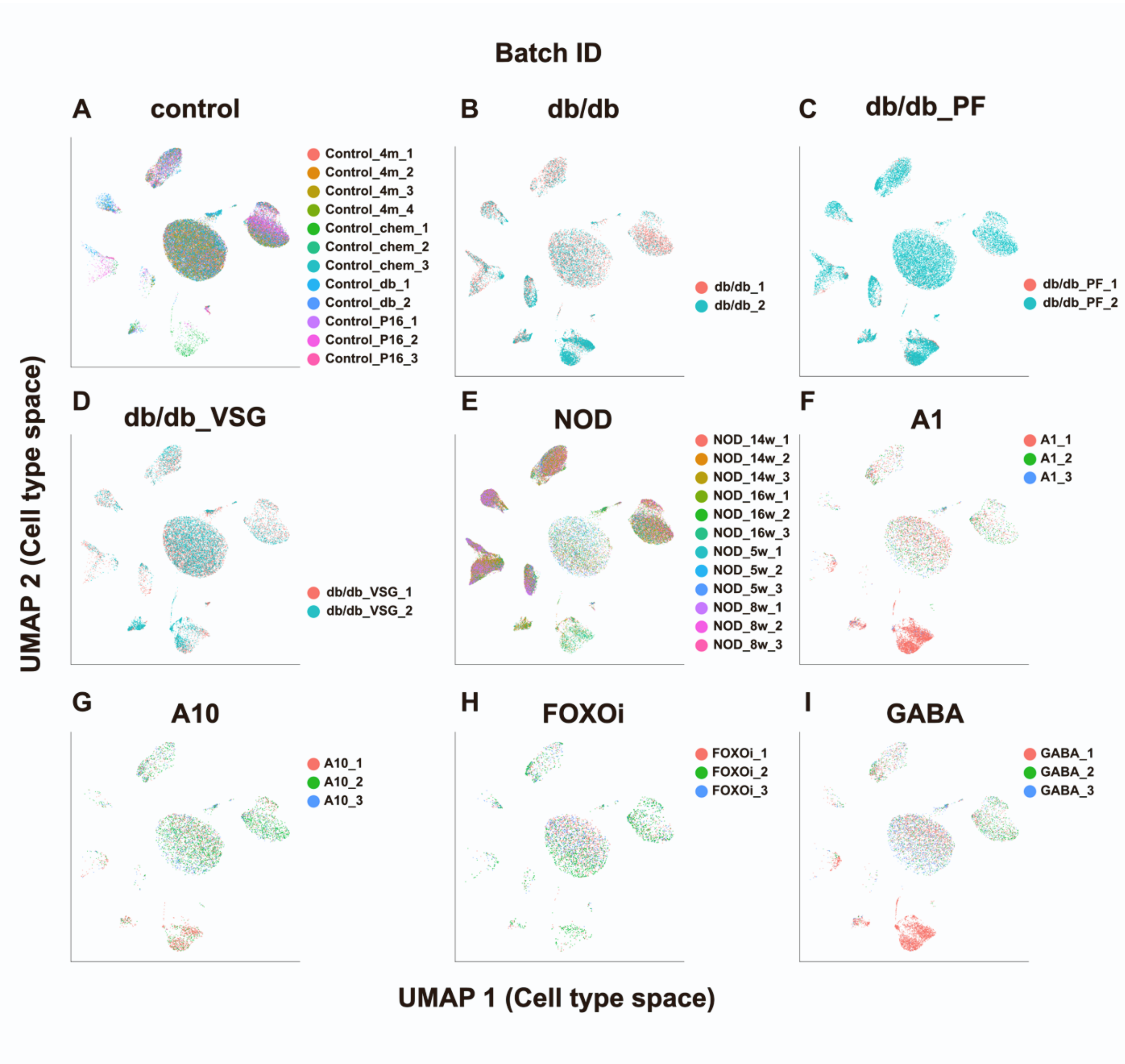

Figure S15: **Batches on latent variables for cell types**
UMAP plots of the mouse pancreatic atlas based on latent variables for cell types. The colors of the dots show batches, and plots were separated by conditions. (A) control, (B) db/db, (C) db/db_PF, (D) db/db_VSG, (E) NOD, (F) A1, (G) A10, (H) FOXOi and (I) GABA
A1 and A10: artemether (1 or 10 μM); FOXOi: FoxO inhibitor

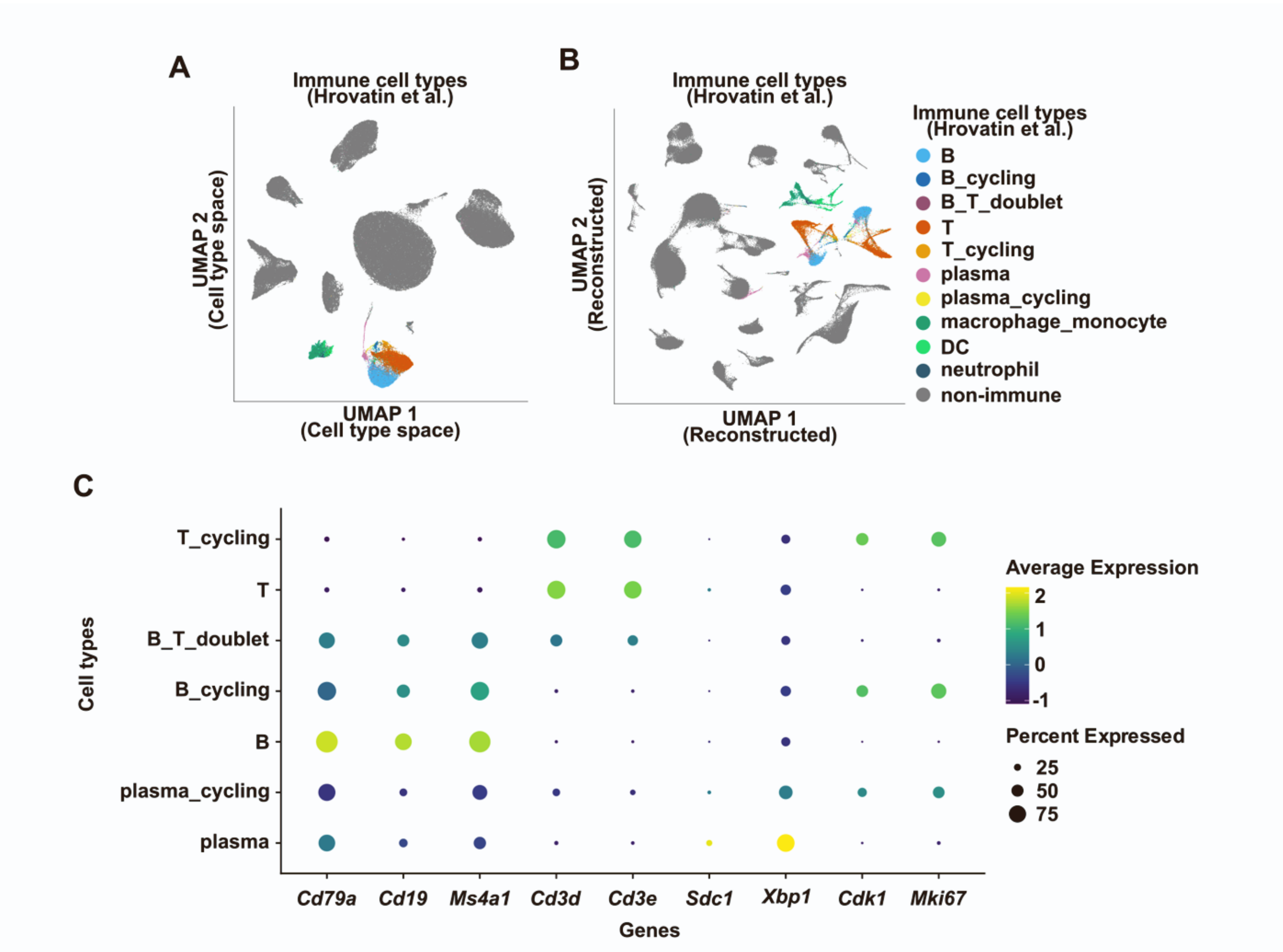

Figure S16: **Separation of lymphoid cells**

(A and B) UMAP plot of the mouse pancreatic atlas based on latent variables for cell types (A) and reconstructed counts (B). The colors of the dots show the annotation of immune cells by Hrovatin et al..

(C) Dot plots of expressions of lymphoid cell types in each cell type. Cell types were based on the annotation of immune cells by Hrovatin et al.. Only lymphoid cells were shown.

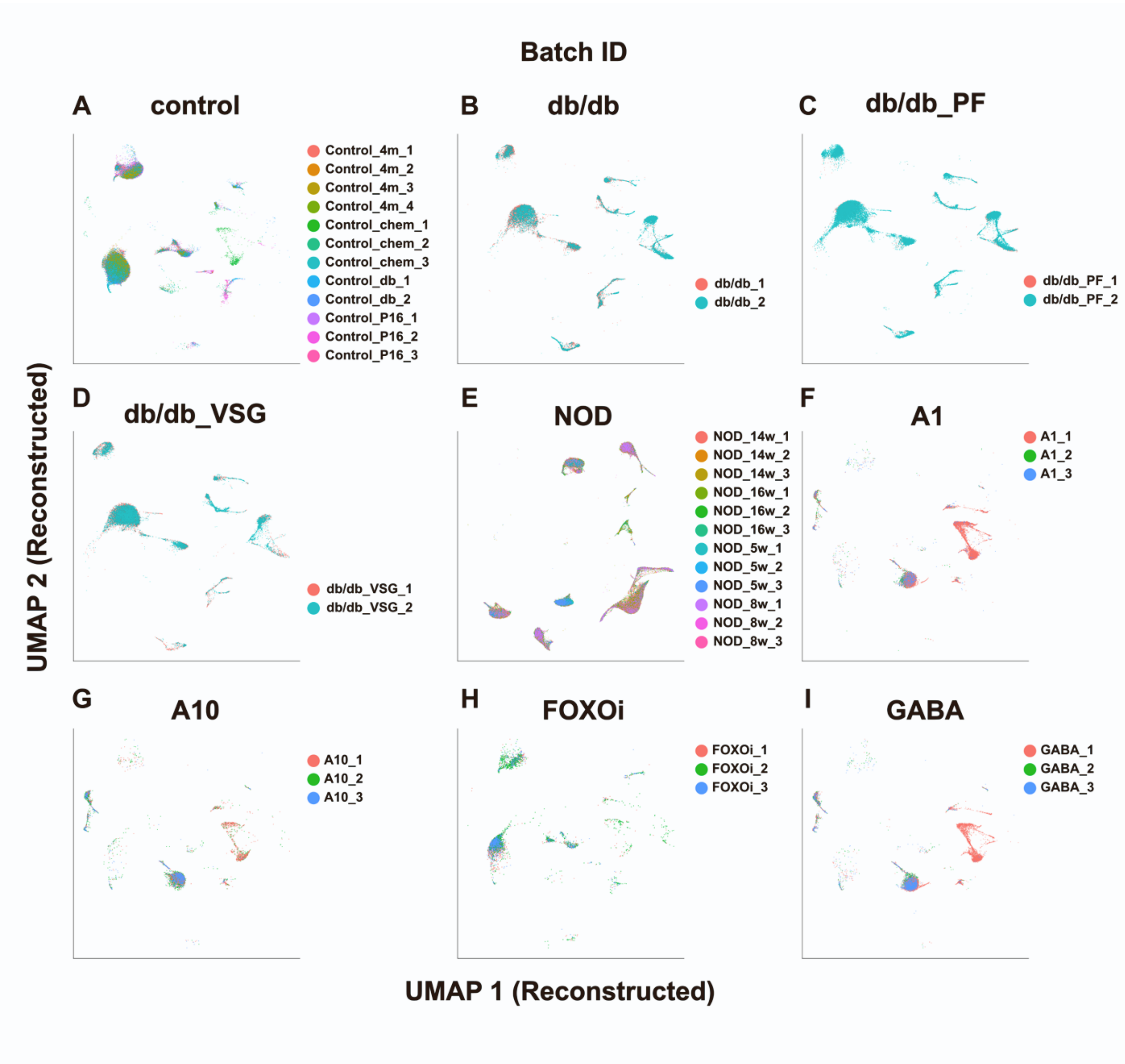

Figure S17: **Batches on reconstructed counts**
UMAP plots of the mouse pancreatic atlas based on reconstructed counts. The colors of the dots show batches, and plots were separated by conditions. (A) control, (B) db/db, (C) db/db_PF, (D) db/db_VSG, (E) NOD, (F) A1, (G) A10, (H) FOXOi and (I) GABA
A1 and A10: artemether (1 or 10 μM); FOXOi: FoxO inhibitor

A

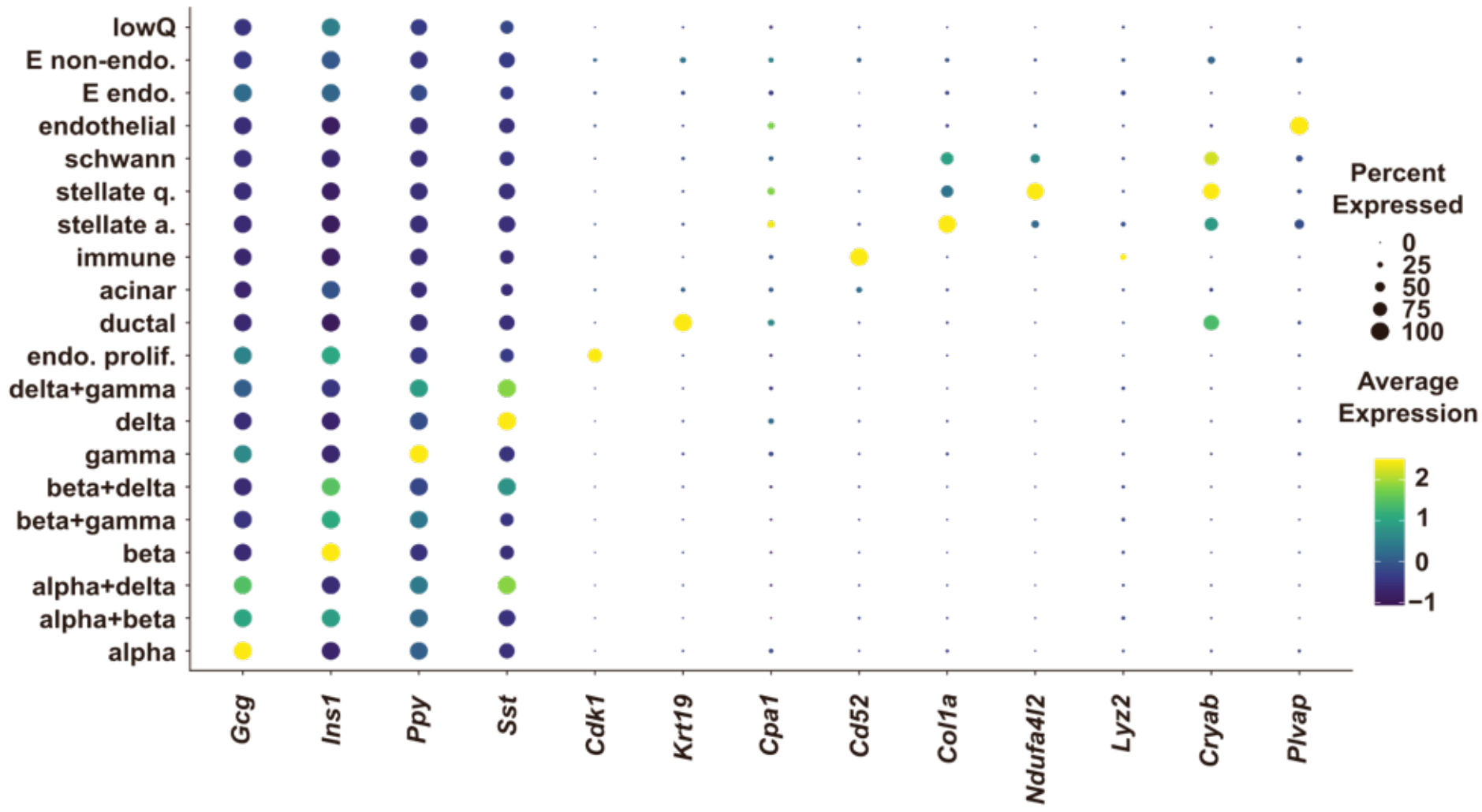

Figure S18: **Reconstructed counts of cell type markers**

Dot plot of the expression marker gene of each cell type shown in Hrovatin et al.

endo.: endocrine; prolif.: proliferating; stellate a. : stellate-activated; stellate q.: stellate-quiescent; low-Q: low quality cell; E: embryonic (misannotated)

$\alpha + \beta$, $\alpha + \delta$, $\beta + \gamma$, $\beta + \delta$ and $\delta + \gamma$ means doublet of two cell types.

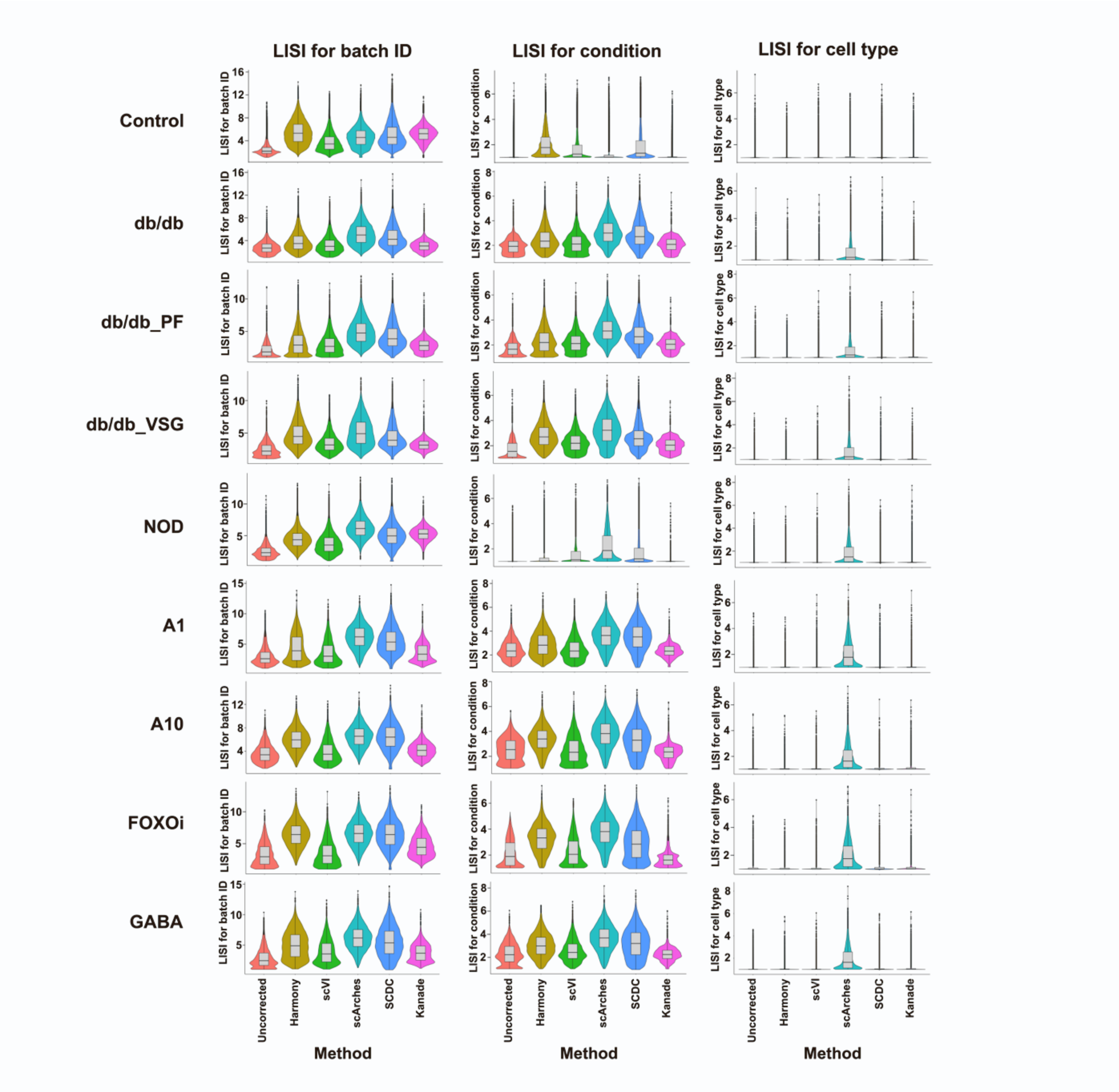

Figure S19: **LISIs of mouse pancreatic dataset**
Violin plots of LISI values for (left) batch ID, (middle) condition, and (right) cell type. In each plot, the x-axis indicates the method used. Results are shown separately for each condition, ordered from top to bottom as follows: control, db/db, db/db_PF, db/db_VSG, NOD, A1, A10, FOXOi, and GABA.
A1 and A10: artemether (1 or 10 μM); FOXOi: FoxO inhibitor

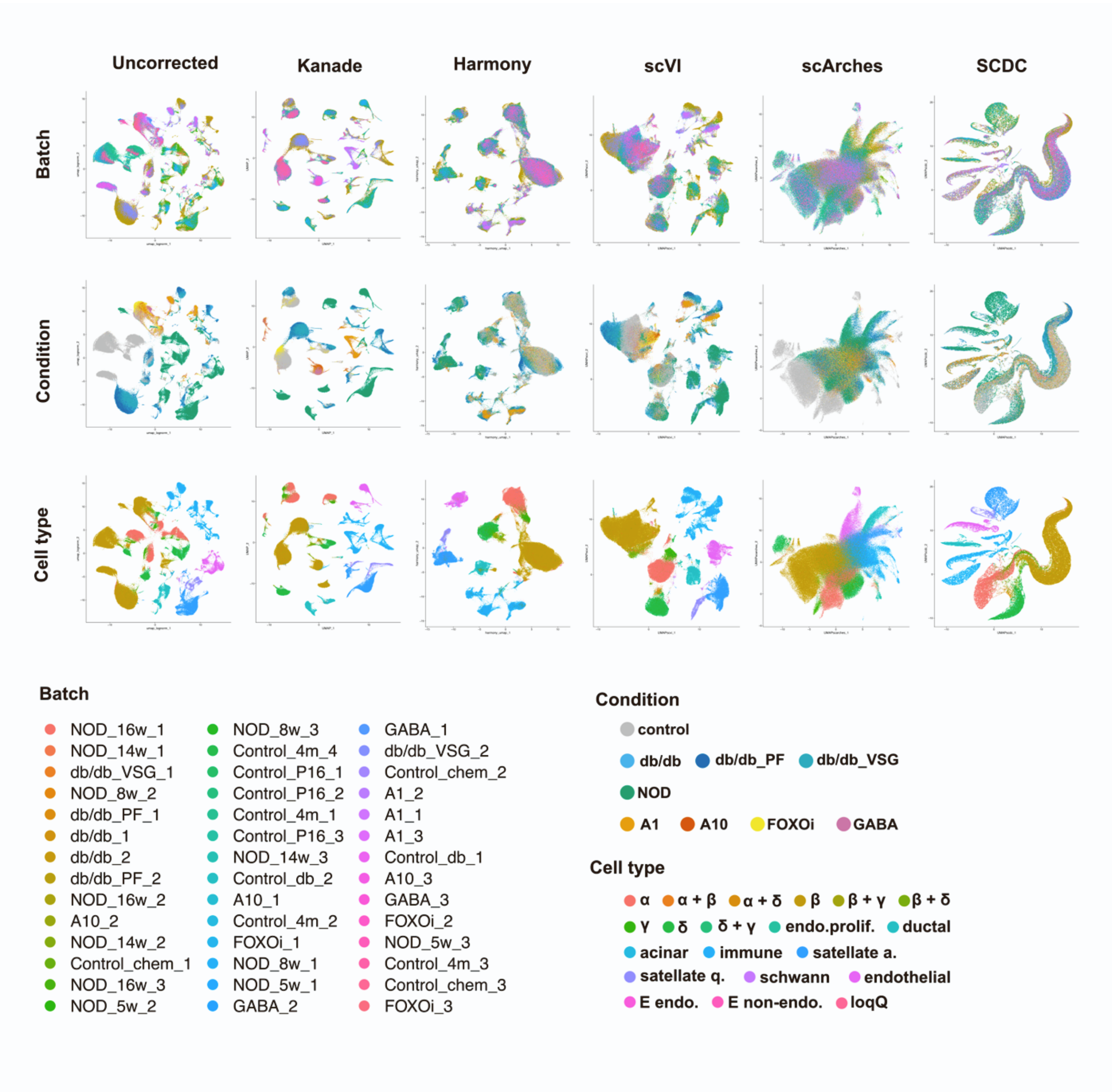

Figure S20: **Comparison UMAP plots of mouse pancreatic dataset among methods** UMAP plot of uncorrected PCA, Kanade-corrected PCA, Harmony, scVI, scArches, and SCDC, respectively from left. From top, colors show Batch ID (donor_id), condition, and cell type, respectively from top.

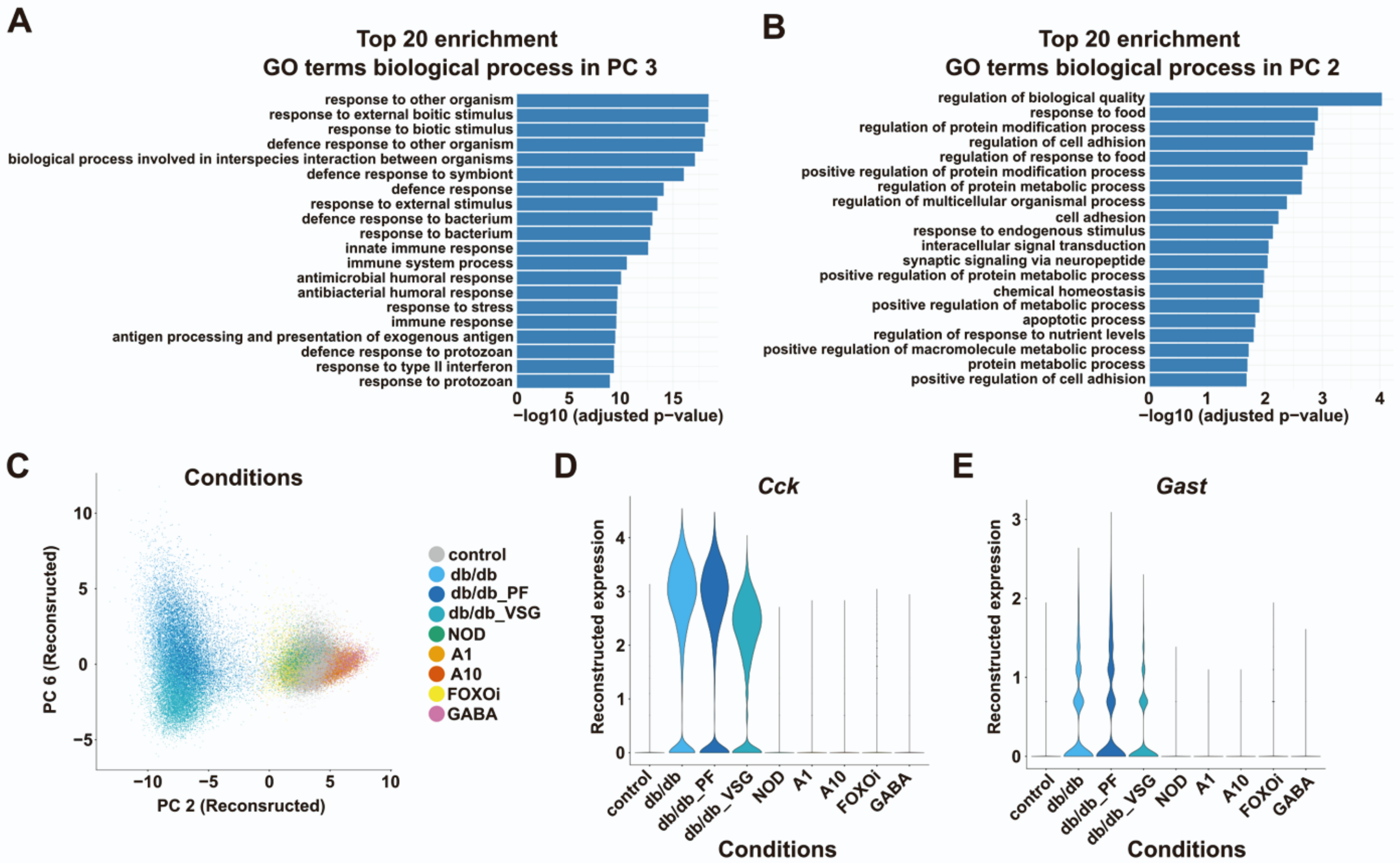

Figure S21: **Conservation of diabetic features in beta cells**
(A and B) Results of GO term enrichment analysis for Top 100 genes negatively contributed to PC 3 (A) and PC 2 (B). Barplots show negative log values of adjusted p-values.
(C) PCA plot of beta cells. The X-axis and the Y-axis represent PC 2 and PC6 respectively. The colors of the dots show the conditions of cells.
(D and E) Violin plots of expression of Cck (D) and Gast (E) in each dataset based on reconstructed counts.
A1 and A10: artemether (1 or 10 μM); FOXOi: FoxO inhibitor

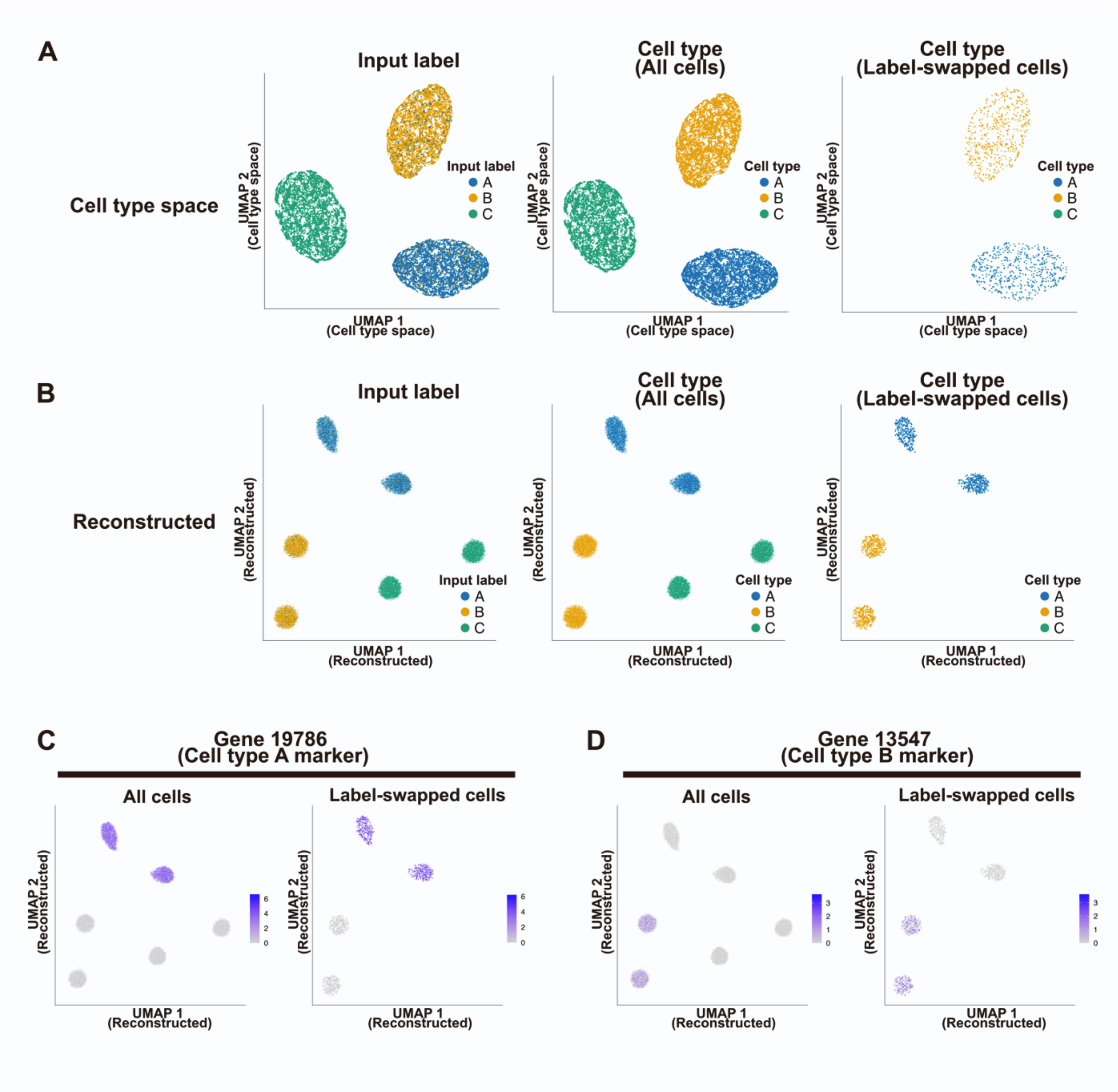

Figure S22: **Robustness of Kanade against label-swapping**
(A) UMAP plots of cell-type space for Label-swapped Data. Colors show (left) input label for training and (middle and right) true cell type, respectively. Only label-swapped cells are shown in the right panel.
(B)UMAP plots of reconstructed space for Label-swapped Data. Colors show (left) input label for training and (middle and right) true cell type, respectively. Only label-swapped cells are shown in the right panel.
(C and D) Expression of (C) Cell type A and (D) Cell type B markers, respectively. Shown on the UMAP of reconstructed count.

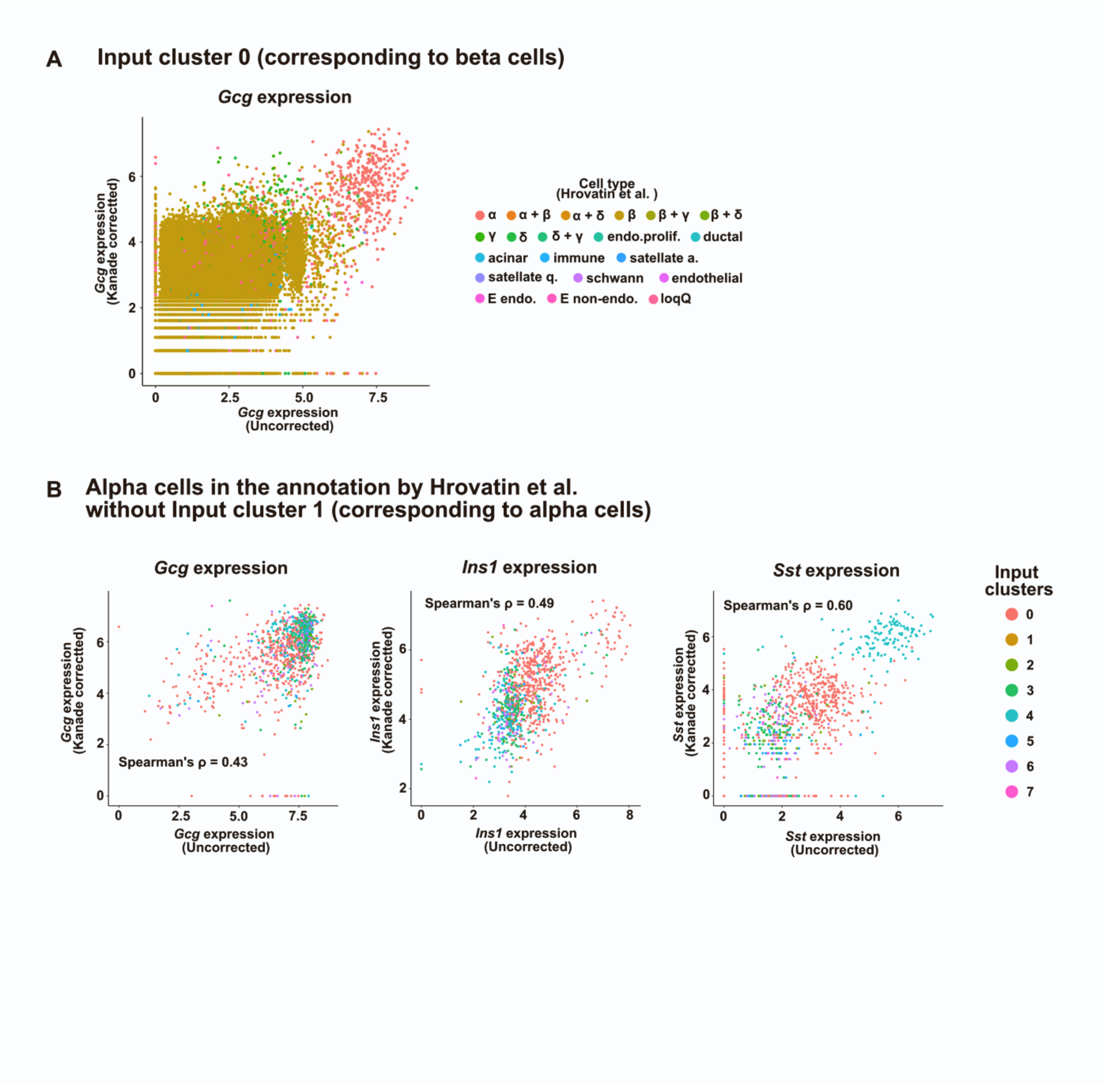

Figure S23: **Reconstruction results of ambiguous annotations**
(A) *Gcg* expression in the input cluster 0. X- and Y- axis represent the expression befor and after correction, respectively. Colors of dots show annotaion of cells by Hrovatin et al.
(B) Expression of marker genes in cells annotated as alpha cells by Hrovatin et al. but assigned to input clusters other than cluster 1. From left, expressions of *Gcg*, *Ins1* and *Sst*, respectively. X- and Y- axis represent the expression befor and after correction, respectively. Colors of dots show input clusters.

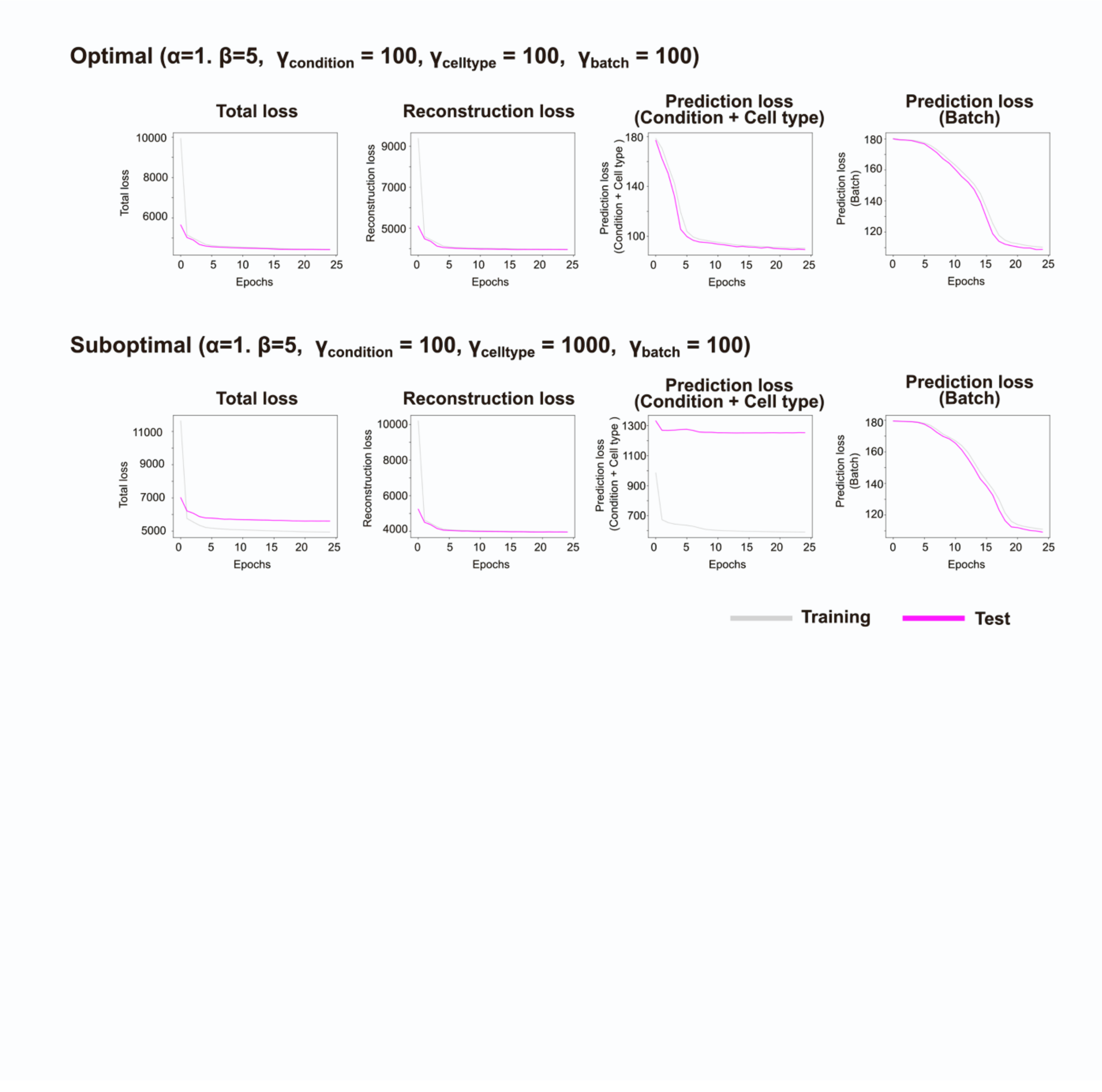

Figure S24: **Example of hyperparameter selection**
Plots of values of loss functions in the training for Discrete Data. From left, total loss, reconstruction loss, prediction loss of disease and cell type, and prediction loss of batch. Top panels show the optimal case with small over-fitting and bottom panels show an example of suboptimal case with large over-fitting.
